# Effect of serum 25-hydroxyvitamin D level on quadriceps strength: a systematic review and meta-analysis

**DOI:** 10.1186/s13102-024-01007-z

**Published:** 2024-10-14

**Authors:** Michael Tim-yun Ong, Kitson Chun-Kit Tsang, Victor Yan Zhe Lu, Stacy Lok Sze Yam, Wei Shen, Gene Chi-Wai Man, Patrick Shu-hang Yung

**Affiliations:** 1 Department of Orthopaedics and Traumatology, Faculty of Medicine, The Chinese University of Hong Kong, Hong Kong SAR, China; 2 School of Clinical Medicine, University of Cambridge, Hills Rd, Cambridge, CB2 0SP UK

**Keywords:** 25-hydroxyvitamin D, Quadriceps strength, Anterior cruciate ligament reconstruction, Meta-analysis

## Abstract

**Background:**

Vitamin D deficiency has been linked to poor muscle function, cartilage degeneration, and the development of knee osteoarthritis. However, the impact of serum 25-hydroxyvitamin D [25(OH)D] level on quadriceps muscle strength remains inconclusive, largely due to variations in study designs, differences in study populations, and the influence of confounding factors such as co-supplementation with other vitamins. The existing literature presents mixed findings, highlighting the need for a comprehensive evaluation of the available evidence.

**Purpose:**

This systematic review and meta-analysis aim to summarise.

**Study design:**

Systematic review; Level of evidence, 4.

**Methods:**

Searches were conducted using Medline (Ovid), Embase (Ovid), CINAHL (EBSCOhost), and SPORTDiscus (EBSCOhost), which aimed to summarise recent (published after 2000 and before March 1st, 2024) studies reporting the effects of serum 25(OH)D levels on quadriceps strength. Appraisal tool for Cross-Sectional Studies (AXIS) for cross-sectional studies and Quality in Prognosis Studies (QUIPS) for longitudinal studies. Results from the AXIS and QUIPS tools were used for GRADE quality assessment. The review was carried out using PRIMSA guidelines and registered in PROSPERO (ID: CRD42022313240).

**Results:**

Four hundred studies were screened and 28 studies with 5752 participants were included. 28 published studies (24 cross-sectional and 4 longitudinal) were identified. Key results supported the significant positive correlation between serum 25(OH)D levels and isokinetic quadriceps strength at 180°/s in elderly and athletic populations with a correlation coefficient of 0.245 (95%CI: 0.078–0.398, *p* = 0.004). However, no significant correlation was found with isometric quadriceps strength or isokinetic strength at 60°/s (r = 0.190, *p* = 0.085). There was only a weak negative correlation with MVC.

**Conclusion:**

This review found a statistically significant positive correlation between serum 25(OH)D levels and isokinetic quadriceps strength. This has important clinical implications, especially in the elderly cohort, with higher 25(OH)D levels being associated with a reduced incidence of falls and fragility fractures.

**Supplementary Information:**

The online version contains supplementary material available at 10.1186/s13102-024-01007-z.

## What is known about the subject

Previous research has established that low serum 25(OH) D level is associated with muscle weakness. But how it negative influences quadricep strength is incomprehensive. This deficiency has been linked to poorer recovery in lower extremity function and an increased rate of revision ACL surgery. However, the broader implications of vitamin D deficiency on muscle strength and recovery outcomes are still not fully understood.

## What this study adds to existing knowledge

Serum 25(OH)D levels showed a statistically significant positive correlation with isokinetic quadriceps strength in elderly and athletic populations.

## Introduction

Vitamin D is a fat-soluble vitamin that has an important role in musculoskeletal health. Vitamin D is either synthesised in the skin after sun exposure or ingested in food, with the former accounting for 80% of vitamin D stores in the body [1]. In both cases, vitamin D is hydroxylated in the liver to 25-hydroxyvitamin D (25(OH)D), and then in the kidneys to its active form, 1,25-dihydroxyvitamin D (calcitriol). Calcitriol activates the vitamin D receptor (VDR) in cells to exert its function. The measurement of serum 25(OH)D levels is a common way to assess vitamin D status. Defined as a 25(OH)D level < 25 nmol/L [2], vitamin D deficiency leads to rickets in children and osteomalacia in adults [2]. There has been a rapidly expanding array of literature discussing the effect of vitamin D deficiency and poor muscle functioning. Studie have shown that a large number of VDRs are expressed in myocytes, allowing the uptake of calcitriol, whose effects are mediated by genomic and non-genomic mechanisms [3].

Patients with severe vitamin D deficiency show muscle atrophy before any signs of osteomalacic bone involvement [4]. Observational studies have shown an association between 25(OH)D levels, muscle strength, and physical function, with most suggesting a positive effect of vitamin D [5–9]. A deficiency in vitamin D has been shown to impair muscle action and lead to sarcopenia as well as decreased muscle strength [10, 11].

Vitamin D is closely related to skeletal muscle function by associated with the large number of VDRs found there [12]. It can regulate its downstream pathways that have been observed to influence the proliferation and differentiation of skeletal muscles and in the inhibition of apoptosis [13]. In addition, vitamin D also affect the diameter and number of type II muscle fibres, which was regarded as faster muscle contraction fibres [14]. It may result in type II muscle atrophy, subsequently influence the performance of short high-power exercises. This is mainly observed in the elderly [15].

Current evidence does not provide a comprehensive understanding of the effect of vitamin D deficiency on quadriceps muscle strength, primarily due to significant variations in study design, study populations, and the presence of confounding factors such as co-supplementation with other vitamins. These inconsistencies make it challenging to draw definitive conclusions. However, given the recent promising findings in the field [16, 17], there is a clear need for a quantitative meta-analysis to consolidate the existing evidence. Such an analysis could offer valuable insights into the pathogenesis and management of knee osteoarthritis (OA) and potentially improve the success rate and recovery outcomes for patients undergoing anterior cruciate ligament reconstruction (ACLR) surgery. This review aims to summarise the available data and establish a clearer relationship between serum 25-hydroxyvitamin D (25(OH)D) levels and quadriceps strength.

## Methods

This review was carried out according to the Preferred Reporting Items for Systematic Reviews and Meta-Analyses (PRISMA) statement protocol and registered in the International prospective register of systematic reviews (PROSPERO) (ID: CRD42022313240) [18].

### Selection criteria

Studies that measured serum concentrations of 25(OH)D as an indicator of vitamin D status were included. Studies using other types of vitamin D indicators such as 1,25- dihydroxyvitamin D or dietary intake of vitamin D were excluded. Studies with participants receiving any form of vitamin D supplementation or exercise training as interventions were excluded.

Studies evaluating the following outcome measures were included:

- Knee isokinetic measurement at any angular velocity
- Quadriceps isometric measurement at any knee flexion angle
- Quadriceps maximal voluntary contraction (MVC)
- Quadriceps muscle size

Studies with functional measurements not specific to quadriceps (or knee extensor muscles) were excluded.

### Search algorithm

Four databases were searched from inception to March 1st 2024. Medline (Ovid), Embase (Ovid), CINAHL (EBSCOhost) and SPORTDiscus (EBSCOhost) without any language or year restrictions. The search contained the following terms and their synonyms: ‘vitamin D’, ‘isometric’, ‘quadriceps’, ‘muscle strength’. The full search is shown in Additional File 1. A snowball search was performed, whereby references of included studies, and studies that cited any of the included studies were also searched. Search results were imported into EndNote™20. After deduplication, two reviewers initially screened the title and abstract of each study. Potentially eligible studies were then retrieved for screening in full text, based on the inclusion and exclusion criteria provided in Additional File 2. A third reviewer was contacted for unresolvable disagreements.

### Data extraction

Data were extracted into tables created in a standardised excel spreadsheet, which were used for evidence synthesis, risk of bias analysis, and quality assessment. From each study, the following data were extracted:

- Study characteristics
- Patient demographics
- Isokinetic measurement, isometric measurement, maximal voluntary contraction, and muscle size

### Data analysis

Quantitative data that were comparable across studies were selected for meta-analysis, such as isokinetic quadriceps strength, isometric quadriceps strength, MVC, and muscle size. Meta-analyses were carried out using MedCalc. As we anticipated considerable between-study heterogeneity, a random-effects model was used. The inverse-variance method was used to pool effect sizes. Where it was not provided, standard deviations were estimated using the Wan et al. [19] estimator, allowing the standard deviation to be estimated form the mean, median, or sample size. Section 7.7.3.3 of the Cochrane Handbook was utilised when estimating the standard deviation from the p-value [20]. Where data was incomplete, the corresponding author of the respective study was contacted by email. Higgins and Thompson’s I^2^ statistic [21] and Cochran’s Q test [22] were used as measures of heterogeneity. Subgroup analyses were performed according to athletic status (athlete vs non-athlete, sample size = 191), mean age (> 60 vs < 60 years, sample size = 371), isokinetic outcomes at 60o/s, and isokinetic outcomes at 180o/s.

### Risk of bias

Two reviewers independently assessed the risk of bias using the Appraisal tool for Cross-Sectional Studies (AXIS) for cross-sectional studies and Quality in Prognosis Studies (QUIPS) for longitudinal studies. The former is a twenty item critical appraisal tool addressing study design as well as risk of bias [23]. The latter consists of items organised into six categories, with each using a three point Likert scale – low, moderate, or high risk of bias [24]. Results from the AXIS and QUIPS tools were used for GRADE quality assessment [25] to judge the quality of evidence on a four-point Likert scale – very low, low, moderate, and high. Randomised controlled studies (RCT) were given an initial rating of ‘high’, whereas non-RCTs were given an initial rating of ‘low’. Five criteria were used to downgrade studies: risk of bias, consistency, directness, precision, and publication bias; three criteria were used to upgrade studies: magnitude of effect, dose response, and effect of confounding factors [26].

## Results

**Fig. 1:**
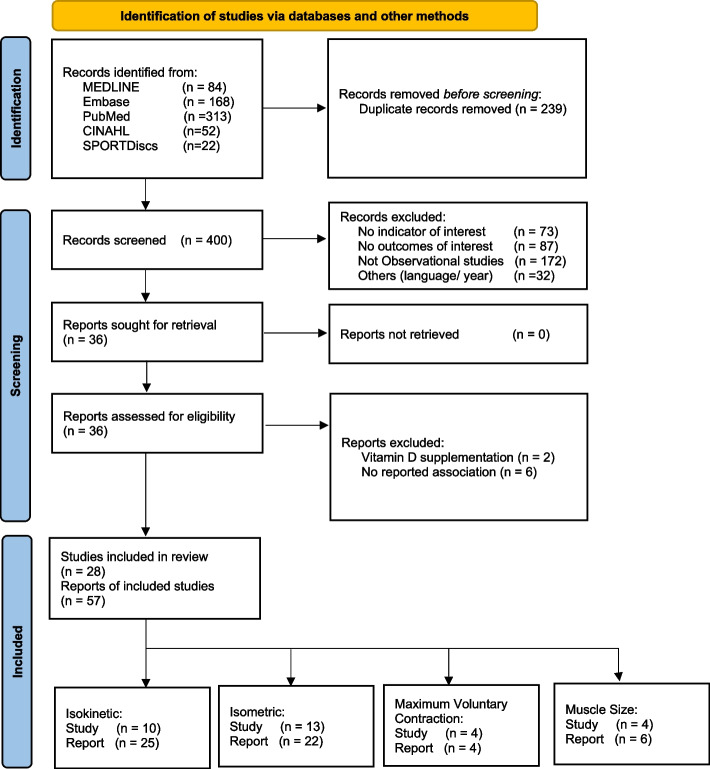
PRISMA flowchart

A total of 639 studies were identified from bibliographic databases. Following de-duplication, 400 studies were included for the title and abstract screening. Full texts were obtained for 36 studies, from which 28 studies were included for data analysis. The snowball search identified 3343 studies, but no further studies were eligible for inclusion. Figure 1 presents the PRISMA flowchart.

### Study characteristics

Twenty-eight studies were published between 2002 and 2021, including 5,752 participants with mean ages ranging from 20.8 to 85.2 years (Additional File 3). Twenty-four (86%) studies were cross-sectional studies, and the remaining were longitudinal studies. The geographical distribution of included studies is as follows: European countries (*n* = 7) [27–33], USA (*n* = 7) [11, 34–39] or UK (*n* = 6) [9, 40–44], followed by Middle East countries (*n* = 4) [45–48], Australia, (*n* = 2) [49, 50] and 1 each from Brazil [51] and Korea [52]. The overall serum 25(OH)D concentration level ranged from 7.7 to 45.43 ng/mL. Results for specific geographical regions were similar: European countries (10.5 – 42.4 ng/mL), USA (16.3—45.43 ng/mL), UK (9.64 – 31.56 ng/mL) and Middle East countries (7.7 – 39.4 ng/mL).

Among the twenty-eight included studies, ten reported IK measurements [11, 32, 33, 39, 45–48, 51, 52], thirteen reported IM measurements [11, 27, 29–31, 35–38, 42, 44, 49, 50], four reported MVC measurements [9, 28, 40, 41], and four reported muscle size measurements [34, 40, 43, 48]. Three studies reported multiple outcome measures [11, 40, 48]. Regarding the model of dynamometer used, 80% of the studies used Biodex 3 [11, 32, 46–48, 51] or 4 [33, 39] (*n* = 8) to measure IK, whilst the remaining two studies used Cybex 770 Norm [45] and CSMI medical solutions [52]. A variety of brands were used for IM measurements, including Biodex (*n* = 1) [11], Cybex (*n* = 1) [29], Good Strength (*n* = 1) [31], TTM Muscular Meter (*n* = 1) [50], Litek Isometric Chair (*n* = 1) [35], Horizontal Plyo-Press (*n* = 1) [37], CSMI Humac Norm (*n* = 2) [42, 44], customised brands [30, 36, 38], and two handhelds versions (model 160 [27] and Lafayette Nicholas Manual Muscle Tester model 01163 [49]). Biodex (*n* = 1) [41], Cybex NORM (*n* = 1) [40] and customised (*n* = 2) [9, 28] equipment were used to measure MVC (Tables 1 and 2).

**Table 1:** Study characteristics

Study	Study Design	Outcome Measures	Study population	Gender	Location	Age (mean ± SD)	Sampling Source	25(OH)D measurement, results
Dhesi et al., 2002	CSS	MVC	*n* = 80	N/A	England	Group 1 (*n* = 20): 77.5 ± 5.4 Group 2 (*n* = 20): 72.4 ± 4.6 Group 3 (*n* = 20): 75.9 ± 5.9 Control (*n* = 20): 74.0 ± 4.2	Mixed	IDS Gamma-B 25OH Immunoassay, Group 1 (fall + < 12 μg/L, *n* = 20): 9.8 ± 2.2 μg/L Group 2 (fall + 12–17 μg/L, *n* = 20): 13.9 ± 1.7 μg/L Group 3 (fall + > 17 μg/L, *n* = 20): 23.6 ± 5.8 μg/L Control (healthy + > 17 μg/L, *n* = 20): 22.8 ± 5.7 μg/L
Zamboni et al., 2002	CSS	IM	*n* = 269	94 M, 175 F	Italy	Men: 71.8 ± 2.1 Women: 71.9 ± 2.4	Community	Radioimmunoassay, Men: 56.5 ± 37.5 nmol/L Women: 39.4 ± 24.1 nmol/L
Annweiler et al., 2009	CSS	MVC	*n* = 440	0 M, 440F	France	80.1 ± 3.5	Community	Radioimmunoassay, 17.4 ± 10.5 ng/mL
Dretakis et al., 2010	CSS	IM	*n* = 48	13 M 35F	Greece	Male: 73.8 ± 5.1 Female: 70.0 ± 4.5	Community	Enzyme Immunoassay Serum IDS OCTEIA 25-OH vitamin D kit, Male: 76.00 ± 34.73 nmol/L Female: 49.11 ± 29.78 nmol/L
Bredella et al., 2011	CSS	MS	*n* = 68	0 M 68F	USA	35.9 ± 6.7	Community	IDS-iSYS Automated Analyser based on Chemiluminescence, 24.1 ± 15.2 ng/mL
Houston et al., 2011	CSS	IM	*n* = 988	351 M 637F		85.2 ± 3.2	Community	LC-TMS, < 20 ng/mL: 30.8% 20—< 30 ng/mL: 35.9% ≥ 30 ng/mL: 33.3%
Marantes et al., 2011	CSS	IM	*n* = 667	311 M 356F	USA	Men: 56.3 ± 18.5 Women: 57.2 ± 17.7	Community	Radioimmunoassay, Men: 23.0 ± 8.2 ng/mL Women: 22.1 ± 10.0 ng/mL
Stockton et al., 2012	CSS	IM	*n* = 45	0 M 45F	Australia	SLE (*n* = 24): 39.6 ± 11.4 Control (*n* = 21): 40.9 ± 13.3	Mixed	LIAISONÕ 25 OH Vitamin D TOTAL Assay by Chemiluminescent Immunoassay Technology, 68.4 ± 22.4 nmol/L
Barker et al., 2013	CSS	IM	*n* = 14	9 M 5F	USA	32.0 ± 1.0	Not described	High Performance-LC, Upon enrolment: 28.0 ± 2.5 ng/mL > 32 ng/mL: 36%; < 32 ng/mL: 64% < 20 ng/mL: 21%; < 10 ng/mL: 7%
Grimaldi et al., 2013	CSS	IK/ IM	*n* = 419	205 M 214F	USA	44.0 ± 16.1	Institutional	Enzyme-Linked Immunosorbent Assay, 33.6 ng/mL
Salacinski et al., 2013	CSS	IM	*n* = 38	18 M 20F	USA	Crohn’s (*n* = 19): 44.2 ± 10.3 Control (*n* = 19): 41.7 ± 11.2	Mixed	Radioimmunoassay (High-Performance LC), High (*n* = 12): 45.4 ± 1.4 ng/mL Low (*n* = 19): 25.3 ± 1.1 ng/mL
Barker et al., 2014	CSS	IK	*n* = 56	25 M 31F	USA	48.0 ± 1.0	Institutional	Chemiluminescent immunoassay, 25.8 ± 1.1 ng/mL
Civelek et al., 2014	CSS	IK	*n* = 49	0 M 49F	Turkey	Median: 64.3 Interquartile range: 59.0—69.5	Community	Shimadzu Prominence High- Performance LC, Deficient (< 20 ng/mL): 49.0% Normal (≥ 20 ng/mL): 51.0%
Hamilton et al., 2014	CSS	IK	*n* = 342	342 M 0F	Qatar	24.4 ± 8.3	Institutional	Chemiluminescent Immunoassay Technology (Liaison® 25-OH Vitamin D total Assay), 20.7 ± 10.8 ng/mL
Rolighed et al., 2014	CSS	IM	*n* = 106	20 M 86F	Denmark	PHPT (*n* = 58): 55.7 – 61.6 Control (*n* = 58): 55.8 – 61.7	Mixed	Isotope Dilution LC-TMS, PHPT: 57.6 nmol/L (53.3 – 61.8) Control: 59.1 nmol/L (52.7 – 65.6)
Salminen et al., 2015	CSS	IM	*n* = 518	79 M 439 F	Finland	72.8 ± 5.7	Community	OCTEIA Immune-Enzymo-Metric Assay, 65.2 ± 17.2 nmol/L
Yumrutepe et al., 2015	CSS	IK	*n* = 147	137 M 10F	Turkey	COPD (*n* = 90): 60.2 ± 7.8 Control (*n* = 57): 58.9 ± 6.4	Mixed	Radio-Immunometric Assay, COPD: 14.5 ± 11.1 ng/mL Control: 16.8 ± 10 ng/mL
Almurdhi et al., 2016	CSS	MVC/ MS	*n* = 40	28 M 12F	UK	T2DM (*n* = 20): 63.1 ± 10.8 Control (*n* = 20): 61.5 ± 6.0	Not described	T2DM: 72.6 ± 43.5 nmol/L Control: 78.9 ± 48.8 nmol/L
Brannstrom et al., 2017	CSS	IK	*n* = 19	0 M 19F	Sweden	15.3 ± 0.7	Institutional	Automatic Immune Analyser, 50.5 ± 12.8 nmol/L
Brech et al., 2017	CSS	IK	*n* = 63	0 M 63F	Brazil	60.6 ± 3.1	Not described	LIAISON® 25OHD Total Assay kit, 24.2 ± 9.2 ng/mL
Kara et al., 2017	CSS	IK/ MS	*n* = 30	3 M 27F	Turkey	Group I (*n* = 15): 44.4 ± 9.4 Group II (*n* = 15): 39.0 ± 9.9	Institutional	Chemiluminescence Microparticle Immunoassay Method, Group I: 9.4 ± 2.5 ng/mL Group II: 20.7 ± 8.3 ng/mL
Jamil et al., 2017	LS	MVC	*n* = 71	30 M 41F	UK	28.6 ± 6.5	Community	Dual TMS, 28.8 ± 20.5 nmol/L
Balogun et al., 2018	LS	IM	*n* = 1033	506 M 527F	Australia	63.0 ± 7.4	Community	Liquid-Phase Radioimmunoassay, 52.6 ± 18.7 nmol/L
Książek et al., 2018	CSS	IK	*n* = 25	25 M 0F	Poland	21.9 ± 9.8	Institutional	Electrochemiluminescence using Elecsys System, 17.4 ± 5.2 ng/mL
Kim et al., 2020	CSS	IK	*n* = 36	36 M 0F	Korea	22.6 ± 3.2	Institutional	High-Performance LC-TMS Detection, 24.7 ± 7.2 ng/mL
Wilson- Barnes et al., 2020	LS	IM	*n* = 47	31 M 16F	UK	Outdoor (*n* = 22): 21.0 ± 1.8 Indoor (*n* = 25): 20.0 ± 1.4	Institutional	LC (nmol/L), Outdoor, autumn: 54.3 ± 25.3; Indoor, autumn: 57.7 ± 22.0; Outdoor, spring: 31.0 ± 17.5; Indoor, spring: 31.0 ± 16.1
Watson et al., 2021	CSS	MS	*n* = 34	15 M 19F	UK	61.0 ± 12.0	Institutional	LC-TMS, Full Cohort: 10.8 ng/mL (7.9 – 18.0)
Wilson- Barnes et al., 2021	LS	IM	*n* = 50	24 M 26F	UK	22.0 ± 3.3	Mixed	LC-TMS, Spring: 46.7 ± 20.9 nmol/L Summer: 63.1 ± 17.3 nmol/L

*LS* longitudinal study, *CSS* cross-sectional study, *IM* isometric, *IK* isokinetic, *MVC* maximal voluntary contraction

**Table 2:** Summary of meta-analyses

*Correlation between serum 25(OH)D levels and:*	No. of Studies	No. of Participants	Correlation Coefficient	95% CI	*p* value	I^2^ value
IK measurement in any angular velocities	5	401	0.261	0.151 to 0.364	< 0.001	15.7%
IM measurement in any angle of knee flexion	4	1550	0.084	-0.103 to 0.265	0.378	84.4%
MVC measurement	2	511	-0.033	-0.120 to 0.054	0.454	0%
IK measurement in athletic populations	3	191	0.229	0.083 to 0.366	0.002	0%
IM measurement in elderly populations	3	1494	0.301	0.160 to 0.429	< 0.001	76.6%
IK measurement at 60°/s	2	122	0.190	-0.027 to 0.390	0.085	26.4%
IK measurement at 180°/s	2	140	0.025	0.078 to 0.398	0.004	0%

*IK* isokinetic, *IM* isometric, *MVC* maximal voluntary contraction, *CI* confidence interval

### Meta-analysis findings

**Fig. 2:**
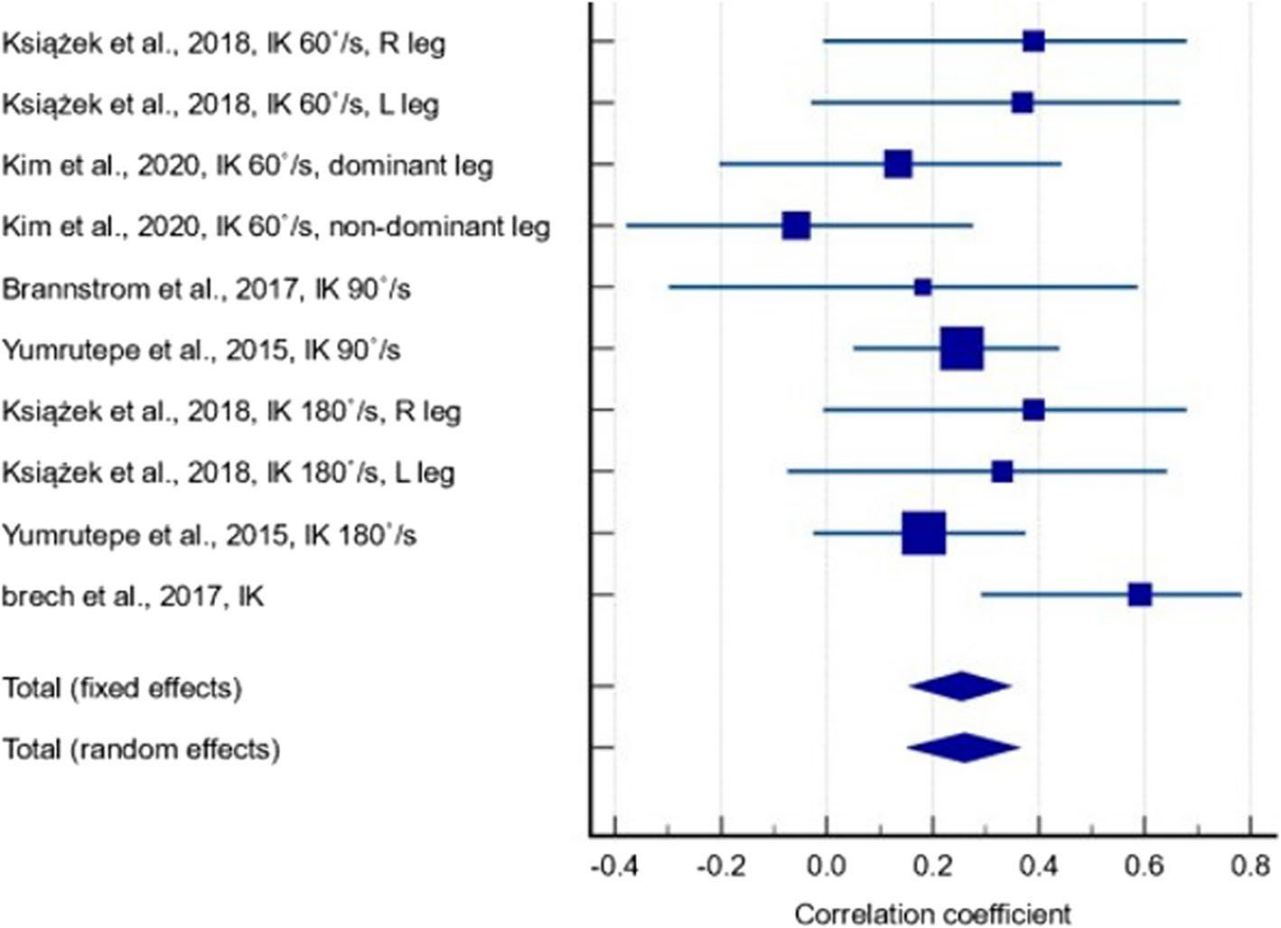
Forest plot showing the correlation between serum 25(OH)D levels and isokinetic measurements at any angular velocity

Out of the 28 included studies, twelve studies [27–30, 32, 33, 37, 41, 47, 50–52] reported the correlation coefficient between serum 25(OH)D levels and muscle strength parameters: isokinetic quadriceps strength (IK); isometric quadriceps muscle strength (IM), and MVC measurements. One study [30] did not report correlation coefficients in a form that could be meta-analysed; the corresponding author was contacted but no response was received. Amongst the remaining eleven studies that were included in the meta-analysis, five studies [32, 33, 47, 51, 52] containing 401 participants reported the correlation between serum 25(OH)D levels and IK measurements at any angular velocity (Fig. 2). In terms of correlation strength, we used standard guidelines where a correlation coefficient (r) between 0.10 and 0.29 is considered weak or low, 0.30 to 0.49 is considered moderate, and values of 0.50 or above are deemed strong [53]. A statistically significant positive correlation was found (r = 0.261, *p* < 0.001, 95% CI: 0.151 – 0.364), with low between-study heterogeneity (I^2^ = 15.7%). The quality of evidence was low since only observational studies were included (Additional File 4). Although a small number of studies in our meta-analysis reported relatively low correlations, it is important to note that the majority of the evidence supports a positive relationship between 25(OH)D and muscle strength across various velocities. This suggests that while the strength of correlation may vary, there remains a consistent trend that supports our conclusion regarding the role of 25(OH)D in quadricep isokinetic strength.

**Fig. 3:**
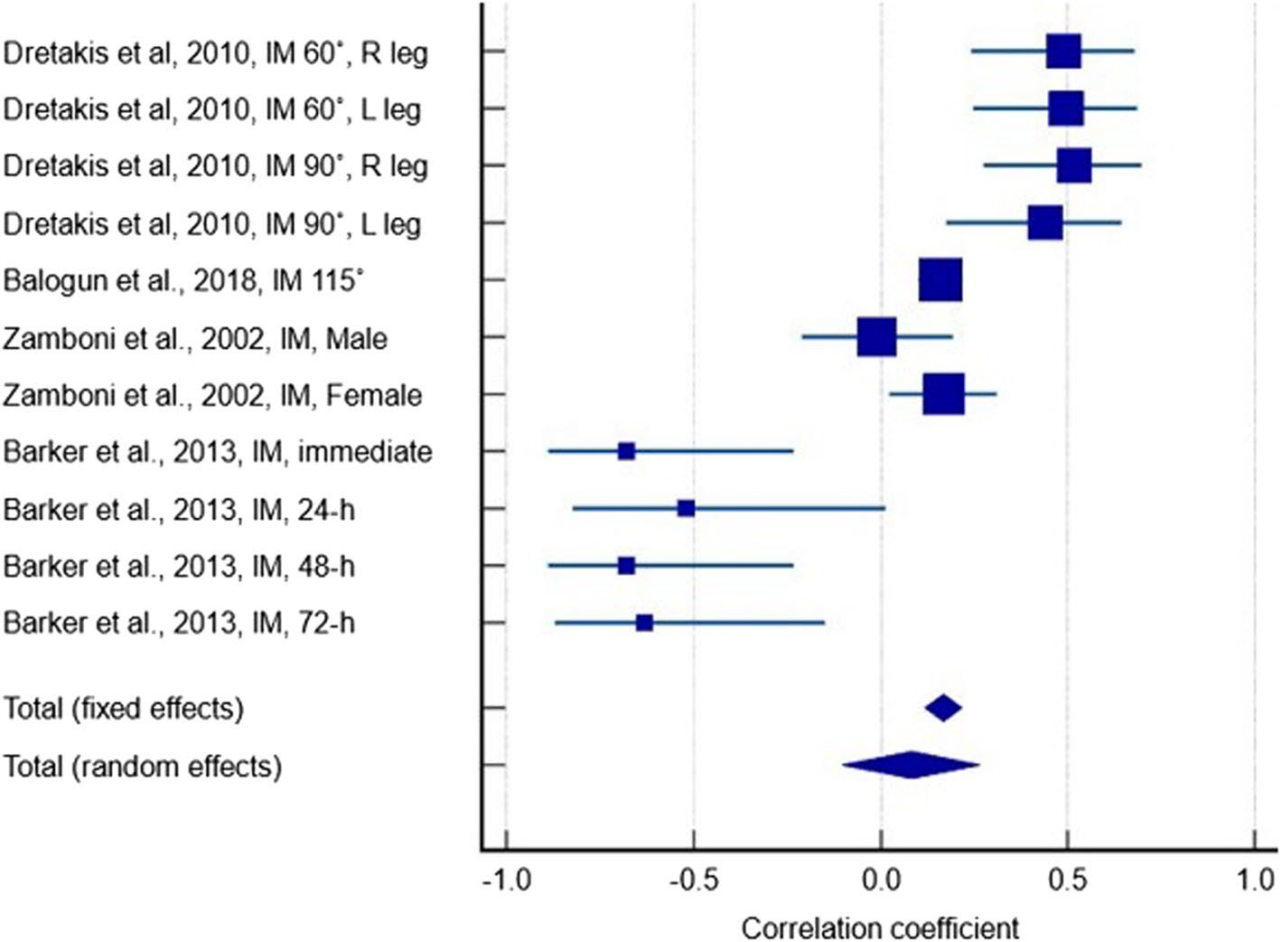
Forest plot showing the correlation between serum 25(OH)D levels and isometric measurements in any knee flexion angle

Four studies [27, 29, 37, 50] containing 1550 participants reported a weak positive correlation between serum 25(OH)D levels and IM measurements in any knee flexion angle (Fig. 3) (r = 0.084, *p* = 0.378, 95% CI: -0.103 – 0.265). The between-study heterogeneity was high (I^2^ = 84.4%) and the quality of evidence was very low due to inconsistency of reported results and significant heterogeneity. There were large variations in effect estimates across studies and effects in opposite directions (i.e. positive and negative correlations).

**Fig. 4:**
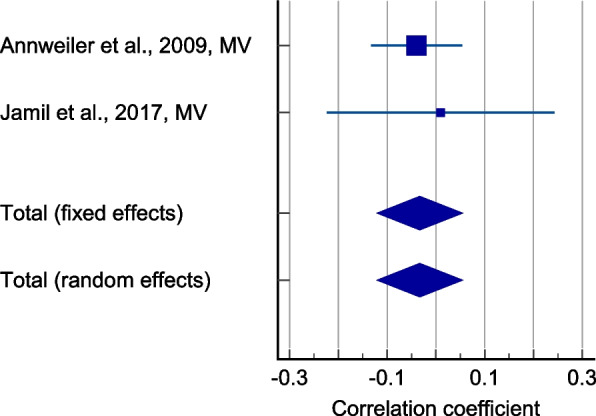
Forest plot showing the correlation between serum 25(OH)D levels and maximum voluntary contraction

Two studies containing 511 participants reported a weak negative correlation between serum 25(OH)D levels and MVC measurements (Fig. 4) (r = -0.033, *p* = 0.454, 95% CI: -0.120 – 0.054). The between-study heterogeneity was low (I^2^ = 0%), however the quality of evidence was very low due to study limitations, imprecision, and possible publication bias.

### Subgroup analyses

**Fig. 5:**
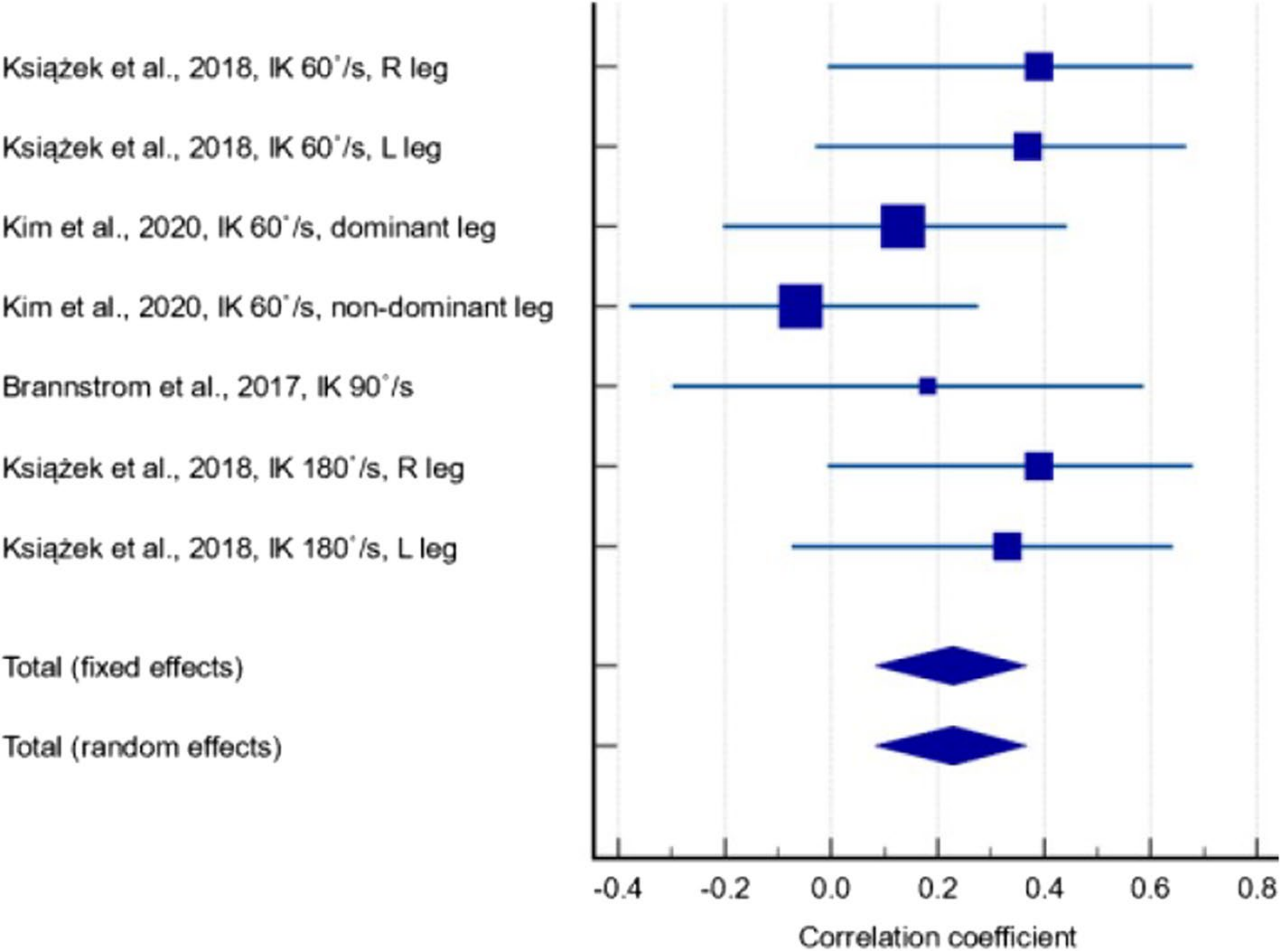
Forest plot showing the correlation between serum 25(OH)D and isokinetic measurements in athletic populations

Regarding studies that reported IK measurements, three [32, 33, 52] recruited elite athletes as participants, including Swedish soccer players at the highest national soccer level from their age categories (with 11.0 ± 2.6 training hours per week) [32], sportsmen from the Polish national Judoist team (with mean career duration 11.5 ± 3.9 years) [33] and members of team Samsung Thunders in the Korean basketball league [52]. Using data from these three studies, a weak-positive correlation was found between serum 25(OH)D and IK measurements (Fig. 5) (r = 0.229, *p* = 0.002, 95% CI: 0.0828 – 0.366). The between-study heterogeneity was low (I^2^ = 15.7%) and quality of evidence was low.

**Fig. 6:**
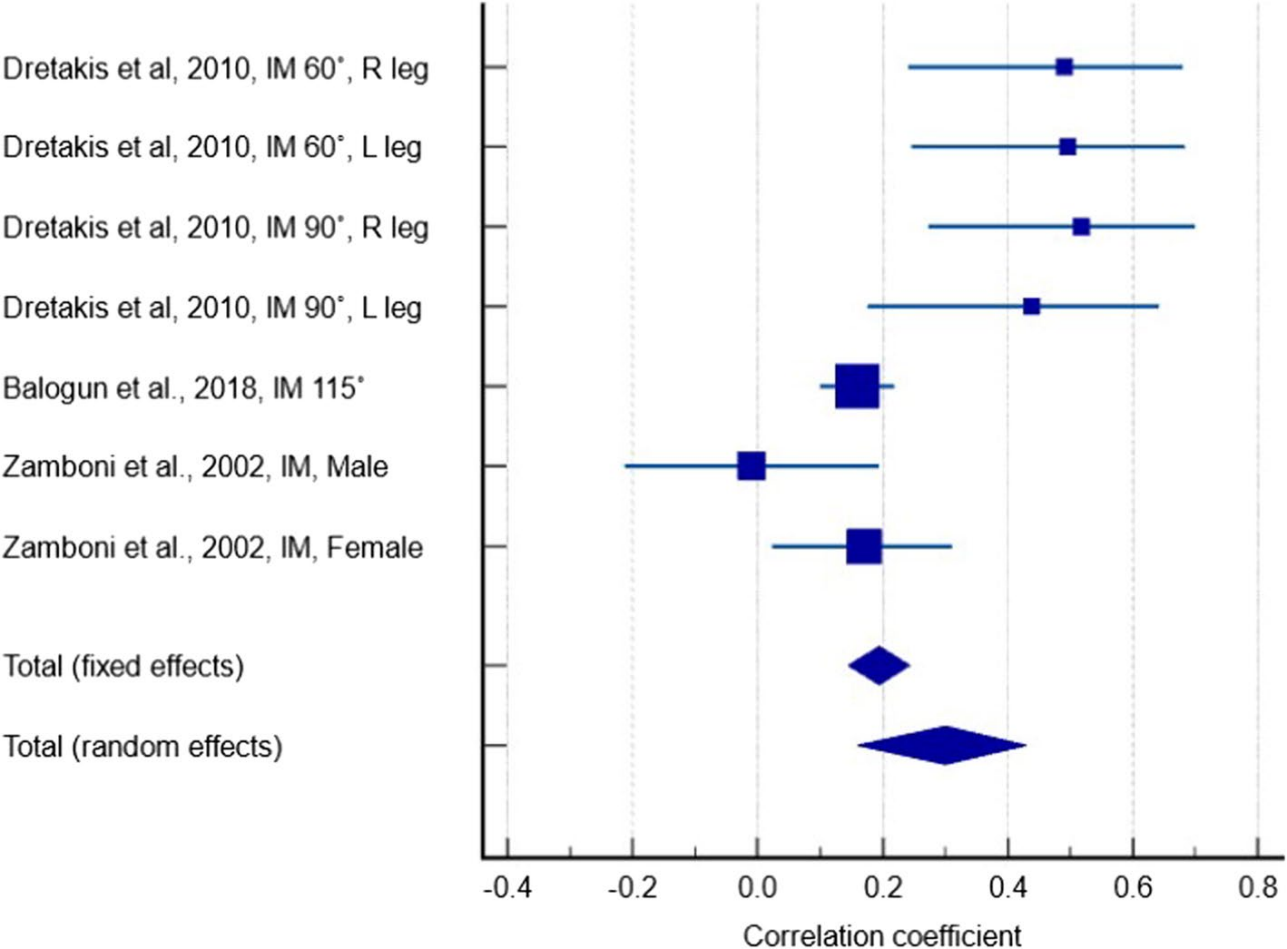
Forest plot showing the correlation between serum 25(OH)D and isometric measurements in the elderly

Amongst the four studies reporting IM measurements included in the meta-analysis, three included participants with a mean age over 60 years old [27, 29, 50]. A moderate positive correlation between 25(OH)D and IM measurements in this age group was found (Fig. 6) (r = 0.301, *p* < 0.001, 95% CI: 0.160 – 0.429). The between-study heterogeneity was high (I^2^ = 76.6%) and the quality of evidence was very low due to inconsistency in the results, publication bias, and significant heterogeneity. There were large variations in the degree to which the outcome was affected.

**Fig. 7:**
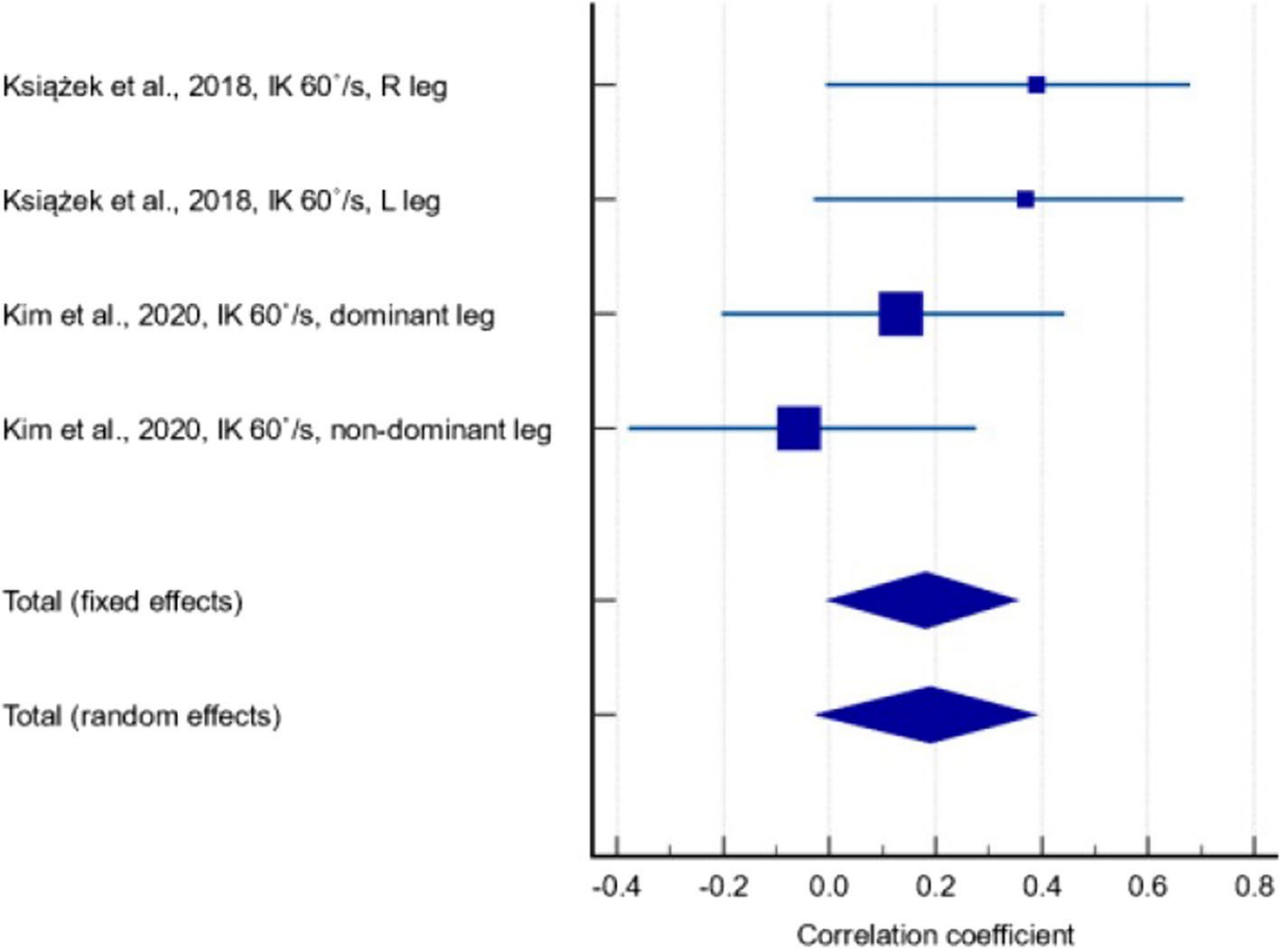
Forest plot showing the correlation between serum 25(OH)D levels and isokinetic measurements at 60°/s

**Fig. 8:**
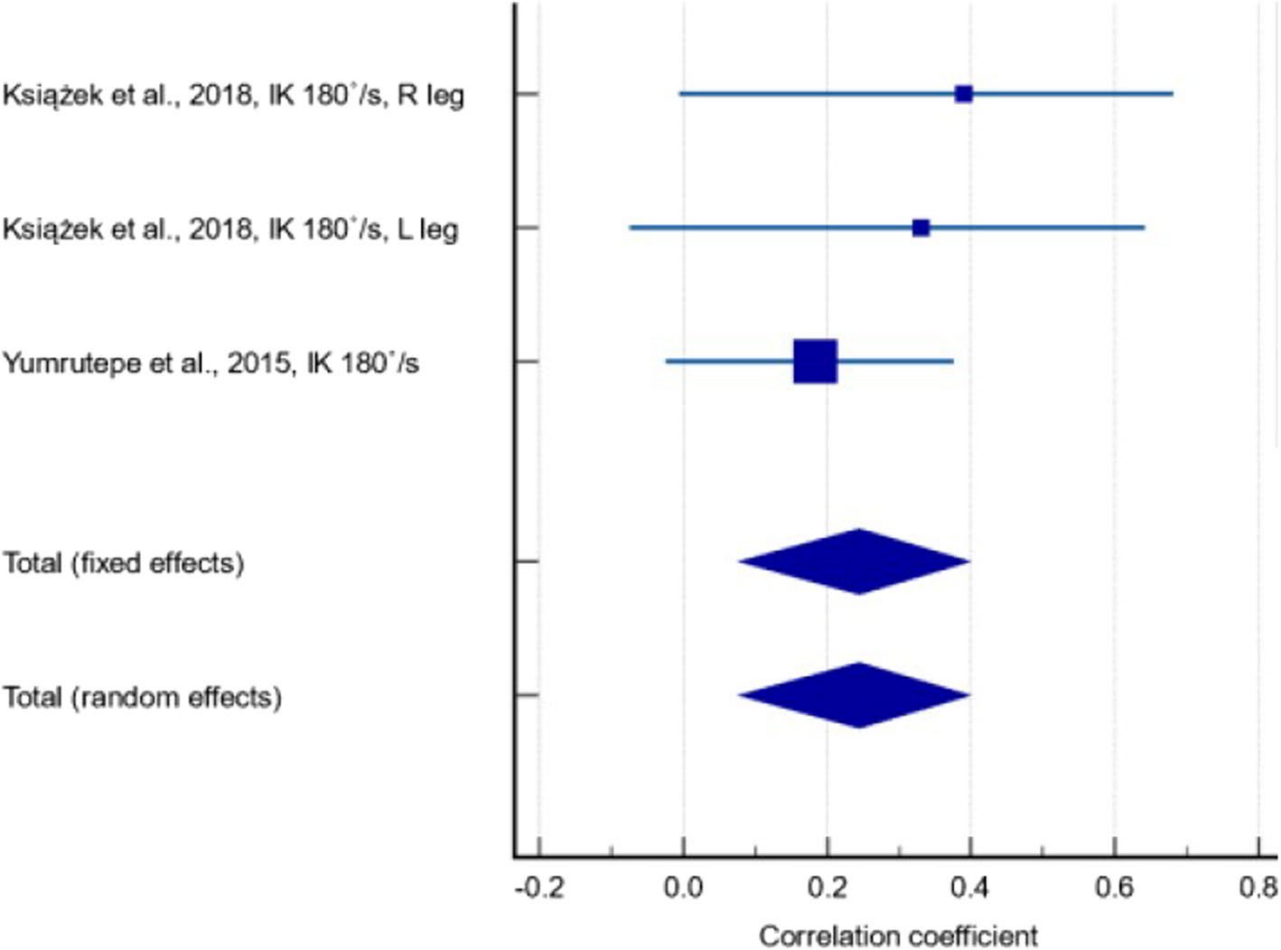
Forest plot showing the correlation between serum 25(OH)D levels and isokinetic measurements at 180°/s

Subgroup analyses were performed for the correlation between serum 25(OH)D levels and IK measurements at 60°/s and 180°/s. The former included results from two studies [33, 52], with a correlation coefficient of 0.190 (Fig. 7) (95% CI: -0.0266 – 0.390; *p* = 0.085). There was low between-study heterogeneity (I^2^ = 26.4%) and the quality of evidence was very low due to inconsistency in the results, with the 95% CI including effects in opposite directions. The latter included results from two studies [33, 47], with a correlation coefficient of 0.245 (Fig. 8) (95% CI: 0.078 – 0.398; *p* = 0.004). There was low between-study heterogeneity (I^2^ = 26.4%) and the quality of evidence was very low due to inconsistency and publication bias.

### Qualitative analysis of studies not included in meta-analysis

Seventeen studies [9, 11, 30, 31, 34–36, 38–40, 42–46, 48, 49] did not report correlation coefficients that could be meta-analysed and therefore were not included in the quantitative analysis. Despite performing statistical analyses between vitamin deficient and control groups, qualitative analyses could not be performed since there is no standardised guideline to compare cohorts with different vitamin D statuses [54–57]. Two studies utilised cohorts with the same 25(OH)D statuses (< 10 ng/mL, 10–20 ng/mL, 20–30 ng/mL, > 30 ng/mL) but reported different outcome measures (IK [46] and IM [42]). Three studies utilised cohorts with similar 25(OH)D statuses [31, 35, 39] (< 20 ng/mL, 20–30 ng/mL, ≥ 30 ng/mL), but were not directly comparable. While most studies included a < 20 ng/ml cohort [31, 35, 39, 42, 44–46], others used the median 25(OH)D value in their study as a cut-off point [48], or a value that has a correlation with serum parathyroid hormone (PTH) secretion [9].

Amongst the seventeen studies not included in the meta-analysis, five studies presented IK measurements [11, 39, 45, 46, 48]. Three of them showed statistically significant differences (*p* < 0.05) between their cohorts [39, 46, 48]. Nine studies presented IM measurements [11, 30, 31, 35, 36, 38, 42, 44, 49], of which five showed statistically significant differences between their cohorts [11, 31, 35, 38, 42]. Grimaldi et al. [11] reported both IK and IM measurements. The remaining four studies presented MVC measurements [9, 34, 40, 43], none of which reported statistically significant findings. Four studies investigated the correlation between serum 25(OH)D levels and muscle size parameters, such as quadriceps cross-sectional area (CSA) [34, 43], quadriceps volume [40, 43], thickness [48], and muscle density [34]. Although all studies reported a positive correlation, none of them were statistically significant.

One study [46] that reported IK measurements recruited an athletic population (members of the Qatar premier “Star” League football division), with measurements performed at 60˚/s and 300˚/s on both legs. Between the four cohorts stratified by serum 25(OH)D levels (< 10 ng/mL, 10- 20 ng/mL, 20–30 ng/mL, and > 30 ng/mL), a statistically significant difference was found in left leg knee concentric extension at 300˚/s (*p* = 0.021). Three studies reporting IK [45] and IM [31, 45] measurements included an elderly population (> 65 years old). Houston et al. [35] utilised two models to report outcome measures. One utilised sociodemographic factor such as age, gender, race, education; the other utilised sociodemographic factors and health behaviours such as alcohol consumption, smoking, and physical activity. The former showed no significant correlation between 25(OH)D levels and quadriceps extensor strength. The latter showed that after adjusting for body weight, quadriceps strength was significantly lower in those with 25(OH)D deficiency (< 20 ng/ml) compared to those with sufficient 25(OH)D (*p* = 0.02) [35]. This suggests 25(OH)D deficiency is associated with poorer muscle quality [35]. Salminen et al. [31] showed greater right (*p* = 0.044) and left (*p* = 0.010) quadriceps strength in those with normal 25(OH)D levels, compared with deficient 25(OH)D levels.

### Risk of bias

Using the AXIS tool for cross-sectional studies (Additional File 5), only three studies justified their sample size [37, 40, 49]. Two studies [40, 46] neither provided an exclusion criteria nor addressed how they dealt with non-responders. Three studies [33, 38, 47] did not specify the statistical test used and the methodology was not sufficiently described to be repeated.

Using the QUIPs tool for prognostic studies (Additional File 6), the overall rating was low for the following domains: study participation, prognostic factor measurement, outcome measure, confounding factors, statistical analysis and reporting.

## Discussion

These findings of this meta-analysis show a statistically significant positive correlation between serum 25(OH)D levels and isokinetic quadriceps strength. The significant positive correlation remained when looking at isokinetic quadriceps strength in elderly populations (> 65 years old), in athletic populations, and isokinetic quadriceps strength at 180°/s. This meta-analysis did not find a significant positive correlation between 25OHD levels and isometric quadriceps strength and isokinetic quadriceps strength at 60°/s. There was a weak negative correlation between 25(OH)D levels and MVC, but this was statistically insignificant.

The results of this review could be important for improving public health since poor lower limb muscle strength is a predictor of functional disability [58], dependence in older people [58], poor quality of life [59], and all-cause mortality [60]. The positive correlation between 25(OH)D and quadriceps strength found in this study could explain the results from meta-analyses suggesting that vitamin D supplementation reduced the risk of an elderly person falling by 14–22% [61–63]. Another meta-analysis reported a three-fold increased risk of recurrent falls in the elderly with lower extremity weakness [64]. Since quadriceps strength is an important predictor of falls [65], vitamin D supplementation can be an inexpensive and safe way to decrease falls and fragility fractures in the elderly, hence reducing healthcare costs [66].

The positive correlation between serum 25(OH)D levels and lower limb muscle strength agrees with other systematic reviews [1, 67, 68]. Vitamin D has a strong regulatory function on skeletal muscle contraction and tone. In a study on vitamin D deficient individuals, vitamin D3 supplementation improved mitochondrial oxidative function in skeletal muscle [69]. Vitamin D acts by binding to VDRs in myocytes, leading to de novo protein synthesis [70]. This relationship has been shown in both human and animal studies [71]. In a study of gluteus medius biopsies, VDR expression was found to decrease with age, leading to a decreased response of the musculature to vitamin D [72]. Studies on genetic polymorphisms in the VDR show a correlation with muscle strength, muscle size, and calcium homeostasis [73], hence affecting the rate of fragility fractures [74]. Mice with whole-body VDR knockout have a shrunken body, decreased muscle mass, and are weak, even if calcium and phosphate levels were kept constant [75]. Nevertheless, some clinical trials [76, 77] and systematic reviews [78, 79] have shown an insignificant relationship between vitamin D supplementation and muscle strength. Perhaps the associations between serum 25(OH)D and muscle functions have one interpretation, while the effects of vitamin D supplementation on muscle functions have another. Although there can be a significant relationship between 25(OH)D and muscle strength, increasing 25(OH)D through supplementation does not necessarily imply increased muscle strength.

The positive correlation between serum 25(OH)D levels with quadriceps strength seen in this meta-analysis might have been affected by confounding factors not controlled for in the included studies. Vitamin D levels are directly affected by sunlight exposure and diet, and indirectly affected by factors such as religion, ethnicity, latitude. Religious attire, increased time spent indoors during winter months can affect sunlight exposure. Whilst studies have shown that vitamin D has a hypertrophic effect on myocytes [43], Zamboni et al. suggested that the beneficial effects of vitamin D are due to its actions on the contractile strength of myocytes rather than size [27]. Furthermore, some studies have shown the effects of vitamin D on neuromuscular coordination, balance, and postural stability, suggested by the presence of VDRs on the human nervous system [80]. This effect on postural stability could be independent from the effect on muscle strength [28].

Although there is a positive correlation between vitamin D levels and lower limb muscle strength, this is not the case with upper limb muscle strength [28, 64, 68]. It is unclear why vitamin D has a differential effect, but the difference in VDR expression is a possible explanation. The lower limb is utilised more than the upper limb during daily load-bearing exercises, and increased neuromuscular modulation in the quadriceps could result in an increased functional response to vitamin D, upregulating VDR expression in the nuclei [72]. Another reason could be a less sensitive handgrip dynamometer that is less able to pick up small but still significant changes in upper limb muscle strength [67]. However, a meta-analysis of RCTs suggested that vitamin D supplementation significantly increased upper (*p* = 0.005) and lower limb strength (*p* = 0.04) [81]. Grimaldi et al. reported stronger and more consistent associations between vitamin D levels and upper limb strength than lower limb strength [11]. This can be explained by the fact that 25(OH)D affects type II muscle fibres which generate more force than type I fibres [52], and a larger percentage of type II fibres is found in the upper limbs compared to the lower limbs [82]. Given the inconclusive and contradictory evidence, further research is needed to ascertain if vitamin D has a differential effect on the upper and lower limbs, and if so, the physiological processes behind it.

A common complication after ACLR surgery is muscle weakness, preventing athletes from returning to full fitness. In a study of eighteen men undergoing ACLR surgery, isometric quadriceps force was greater in those with a higher baseline 25(OH)D level [83]. This could be due to the presence of IFNγ after surgery, which aids the conversion of 25(OH)D to 1,25-(OH)_2_D [83], the biologically active form of vitamin D. Low vitamin D levels could hinder strength recovery during the inflammatory phase straight after ACLR surgery. Thus, monitoring vitamin D levels, or rather the 1,25-(OH)_2_D to 25(OH)D ratio, can be a simple yet effective rehabilitation method after ACLR surgery.

It is unclear if there is an optimal vitamin D level for muscle function and strength. Despite studies showing a decreased risk of falls and increased lower limb function with vitamin D supplementation [11, 61, 62], it may not be beneficial in certain situations, especially in high doses. In a study of 2256 elderly women receiving 500,000 IU of cholecalciferol annually, the incidence risk ratio of fragility fractures was 1.26 versus the placebo group [84]. An increase in fracture incidence after high dose vitamin D treatment was also seen in Smith et al. whose cohort received 300,000 IU of ergocalciferol annually [85]. This seemingly contradicts the result in Trivedi et al. who reported a 0.78 relative risk compared with placebo for any fracture [86]. However, the dosing regimen differed; Trivedi et al. used 100,000 IU cholecalciferol every four months for five years, whilst Smith et al. used 300,000 IU ergocalciferol annually over three years. Perhaps the large annual dose and subsequent decrease in levels, rather than a large dose per se, is detrimental to fracture prevention [84].

### Strengths and limitations

Methodological strengths of this review include the adherence to the PRIMSA statement, and a rigorous assessment of quality of evidence using the Cochrane GRADE guidelines. The main limitation of this review is the inclusion of observational studies that have a low quality of evidence. The included studies were heterogeneous, with small and unevenly distributed sample sizes, and a variety of dynamometers used. This could have contributed to the significant heterogeneity seen in some analyses, which was mitigated by using a random-effects model. Using Egger’s and Begg’s test, no evidence of publication bias was found. Despite a rigorous attempt to include all suitable studies, one from Turkey were excluded as only the abstract was available in English [87]. This study included studies that only used serum 25OHD levels to indicate vitamin D status, which increases the chance for selective reporting. In over half the included studies, no correlation coefficient between 25OHD and muscle strength were reported, and different vitamin D cohorts were used, preventing a quantitative comparison between studies.

## Conclusion

This review identified a statistically significant positive correlation between serum 25(OH)D levels and isokinetic quadriceps strength, indicating that higher serum 25(OH)D levels may enhance quadricep strength. While there was also a positive correlation between serum 25(OH)D levels and isometric quadriceps strength, this relationship was not statistically significant. These findings suggest that maintaining adequate vitamin D levels could be crucial in preserving muscle strength, particularly in the elderly, who are at a higher risk of falls and fragility fractures. Clinically, this underscores the potential of vitamin D as a preventive measure against such risks. Given the widespread public interest in vitamin D, further research is necessary to establish the optimal serum levels and determine the most effective dosing strategies, including the appropriate mode and duration of vitamin D supplementation.

## Supplementary Information

Supplementary Material 1.

Supplementary Material 2.

Supplementary Material 3.

Supplementary Material 4.

Supplementary Material 5.

Supplementary Material 6.

## Data Availability

The datasets used and/or analyzed during the current study are available from the corresponding author on reasonable request.

## Abbreviations

25OHD: 25-Hydroxyvitamin D
VDR: Vitamin D receptor
PTH: Parathyroid hormone
MVC: Maximal voluntary contraction
AXIS: Appraisal tool for Cross-Sectional Studies
QUIPS: Quality in Prognosis Studies
RCT: Randomised controlled studies
IK: Isokinetic
IM: Isometric
LS: Longitudinal study
CSS: Cross-sectional study

## References

[1] Rejnmark L. Effects of vitamin d on muscle function and performance: a review of evidence from randomized controlled trials. Ther Adv Chronic Dis. 2011;2(1):25–37. 23251739 10.1177/2040622310381934 PMC3513873

[2] Pearce SH, Cheetham TD. Diagnosis and management of vitamin D deficiency. BMJ. 2010;340:b5664. 20064851 10.1136/bmj.b5664

[3] Molina P, Carrero JJ, Bover J, Chauveau P, Mazzaferro S, Torres PU. Vitamin D, a modulator of musculoskeletal health in chronic kidney disease. J Cachexia Sarcopenia Muscle. 2017;8(5):686–701. 28675610 10.1002/jcsm.12218 PMC5659055

[4] Glerup H, Mikkelsen K, Poulsen L, Hass E, Overbeck S, Andersen H, Charles P, Eriksen EF. Hypovitaminosis D myopathy without biochemical signs of osteomalacic bone involvement. Calcif Tissue Int. 2000;66(6):419–24. 10821877 10.1007/s002230010085

[5] Pfeifer M, Begerow B, Minne HW, Suppan K, Fahrleitner-Pammer A, Dobnig H. Effects of a long-term vitamin D and calcium supplementation on falls and parameters of muscle function in community-dwelling older individuals. Osteoporos Int. 2009;20(2):315–22. 18629569 10.1007/s00198-008-0662-7

[6] Grieger JA, Nowson CA, Jarman HF, Malon R, Ackland LM. Multivitamin supplementation improves nutritional status and bone quality in aged care residents. Eur J Clin Nutr. 2009;63(4):558–65. 18043700 10.1038/sj.ejcn.1602963

[7] Bunout D, Barrera G, Leiva L, Gattas V, de la Maza MP, Avendaño M, Hirsch S. Effects of vitamin D supplementation and exercise training on physical performance in Chilean vitamin D deficient elderly subjects. Exp Gerontol. 2006;41(8):746–52. 16797903 10.1016/j.exger.2006.05.001

[8] Gallagher JC. The effects of calcitriol on falls and fractures and physical performance tests. J Steroid Biochem Mol Biol. 2004;89–90(1–5):497–501. 15225827 10.1016/j.jsbmb.2004.03.059

[9] Dhesi JK, Bearne LM, Moniz C, Hurley MV, Jackson SH, Swift CG, Allain TJ. Neuromuscular and psychomotor function in elderly subjects who fall and the relationship with vitamin D status. J Bone Miner Res. 2002;17(5):891–7. 12009020 10.1359/jbmr.2002.17.5.891

[10] Foo LH, Zhang Q, Zhu K, Ma G, Hu X, Greenfield H, Fraser DR. Low vitamin D status has an adverse influence on bone mass, bone turnover, and muscle strength in Chinese adolescent girls. J Nutr. 2009;139(5):1002–7. 19321588 10.3945/jn.108.102053

[11] Grimaldi AS, Parker BA, Capizzi JA, Clarkson PM, Pescatello LS, White MC, Thompson PD. 25(OH) vitamin D is associated with greater muscle strength in healthy men and women. Med Sci Sports Exerc. 2013;45(1):157–62. 22895376 10.1249/MSS.0b013e31826c9a78 PMC3544152

[12] Ceglia L, Harris SS. Vitamin D and its role in skeletal muscle. Calcif Tissue Int. 2013;92(2):151–62. 22968766 10.1007/s00223-012-9645-y

[13] Ceglia L. Vitamin D and its role in skeletal muscle. Curr Opin Clin Nutr Metab Care. 2009;12(6):628–33. 19770647 10.1097/MCO.0b013e328331c707 PMC2901845

[14] Ksiazek A, Zagrodna A, Slowinska-Lisowska M, Vitamin D. skeletal muscle function and athletic performance in athletes-A narrative review. Nutrients. 2019;11(8):1800. 31382666 10.3390/nu11081800 PMC6722905

[15] Todd J, Madigan S, Pourshahidi K, McSorley E, Laird E, Healy M, Magee P. Vitamin D status and supplementation practices in elite irish athletes: an update from 2010/2011. Nutrients. 2016;8(8):485. 27517954 10.3390/nu8080485 PMC4997398

[16] Javadian Y, Adabi M, Heidari B, Babaei M, Firouzjahi A, Ghahhari BY, Hajian-Tilaki K. Quadriceps muscle strength correlates with serum vitamin D and knee pain in knee Osteoarthritis. Clin J Pain. 2017;33(1):67–70. 26889621 10.1097/AJP.0000000000000358

[17] Heidari B, Javadian Y, Babaei M, Yousef-Ghahari B. Restorative effect of vitamin D deficiency on knee pain and quadriceps muscle strength in knee osteoarthritis. Acta Med Iran. 2015;53(8):466–70. 26545990

[18] Page MJ, McKenzie JE, Bossuyt PM, Boutron I, Hoffmann TC, Mulrow CD, Shamseer L, Tetzlaff JM, Akl EA, Brennan SE: The PRISMA, et al. statement: An updated guideline for reporting systematic reviews. The BMJ. 2020;2021:372. 10.1136/bmj.n71 PMC8005924 33782057

[19] Wan X, Wang W, Liu J, Tong T. Estimating the sample mean and standard deviation from the sample size, median, range and/or interquartile range. BMC Med Res Methodol. 2014;14:1–13. 25524443 10.1186/1471-2288-14-135 PMC4383202

[20] Cochrane handbook for systematic reviews of interventions. London: The Cochrane Collaboration [https://handbook-5-1.cochrane.org/chapter_7/7_7_3_3_obtaining_standard_deviations_from_standard_errors.htm#:~:text=Standard%20deviations%20can%20be%20obtained,value%20or%20the%20P%20value.

[21] Higgins JPT, Thompson SG. Quantifying heterogeneity in a meta-analysis. Stat Med. 2002;21:1539–58. 12111919 10.1002/sim.1186

[22] Cochran WG. Some Methods for Strengthening the Common χ 2 Tests. Biometrics. 1954;10(4):417–51.

[23] Downes MJ, Brennan ML, Williams HC, Dean RS. Development of a critical appraisal tool to assess the quality of cross-sectional studies (AXIS). BMJ Open. 2016;6(12):e011458. 27932337 10.1136/bmjopen-2016-011458 PMC5168618

[24] Hayden JA, van der Windt DA, Cartwright JL, Côté P, Bombardier C. Assessing bias in studies of prognostic factors. Ann Intern Med. 2013;158(4):280–6. 23420236 10.7326/0003-4819-158-4-201302190-00009

[25] Guyatt GH, Oxman AD, Kunz R, Vist GE, Falck-Ytter Y, Schünemann HJ. What is “quality of evidence” and why is it important to clinicians? BMJ. 2008;336(7651):995–8. 18456631 10.1136/bmj.39490.551019.BE PMC2364804

[26] How to GRADE https://opal.latrobe.edu.au/articles/journal_contribution/How_to_GRADE/6818894.

[27] Zamboni M, Zoico E, Tosoni P, Zivelonghi A, Bortolani A, Maggi S, Di Francesco V, Bosello O. Relation between vitamin D, physical performance, and disability in elderly persons. J Gerontol A Biol Sci Med Sci. 2002;57(1):M7–11. 11773206 10.1093/gerona/57.1.m7

[28] Annweiler C, Beauchet O, Berrut G, Fantino B, Bonnefoy M, Herrmann FR, Schott AM. Is there an association between serum 25-hydroxyvitamin D concentration and muscle strength among older women? Results from baseline assessment of the EPIDOS study. J Nutr Health Aging. 2009;13(2):90–5.19214335 10.1007/s12603-009-0013-1

[29] Dretakis OE, Tsatsanis C, Fyrgadis A, Drakopoulos CG, Steriopoulos K, Margioris AN. Correlation between serum 25-hydroxyvitamin D levels and quadriceps muscle strength in elderly cretans. J Int Med Res. 2010;38(5):1824–34. 21309499 10.1177/147323001003800530

[30] Rolighed L, Amstrup AK, Jakobsen NF, Sikjaer T, Mosekilde L, Christiansen P, Rejnmark L. Muscle function is impaired in patients with “asymptomatic” primary hyperparathyroidism. World J Surg. 2014;38(3):549–57. 24101026 10.1007/s00268-013-2273-5

[31] Salminen M, Saaristo P, Salonoja M, Vaapio S, Vahlberg T, Lamberg-Allardt C, Aarnio P, Kivelä SL. Vitamin D status and physical function in older Finnish people: A one-year follow-up study. Arch Gerontol Geriatr. 2015;61(3):419–24. 26321481 10.1016/j.archger.2015.08.014

[32] Brännström A, Yu JG, Jonsson P, Åkerfeldt T, Stridsberg M, Svensson M. Vitamin D in relation to bone health and muscle function in young female soccer players. Eur J Sport Sci. 2017;17(2):249–56. 27633075 10.1080/17461391.2016.1225823

[33] Książek A, Dziubek W, Pietraszewska J, Słowińska-Lisowska M. Relationship between 25(OH)D levels and athletic performance in elite Polish judoists. Biol Sport. 2018;35(2):191–6. 30455548 10.5114/biolsport.2018.74195 PMC6234302

[34] Bredella MA, Torriani M, Ghomi RH, Thomas BJ, Brick DJ, Gerweck AV, Harrington LM, Breggia A, Rosen CJ, Miller KK. Determinants of bone mineral density in obese premenopausal women. Bone. 2011;48(4):748–54. 21195217 10.1016/j.bone.2010.12.011 PMC3073669

[35] Houston DK, Tooze JA, Davis CC, Chaves PH, Hirsch CH, Robbins JA, Arnold AM, Newman AB, Kritchevsky SB. Serum 25-hydroxyvitamin D and physical function in older adults: the Cardiovascular Health Study All Stars. J Am Geriatr Soc. 2011;59(10):1793–801. 22091492 10.1111/j.1532-5415.2011.03601.x PMC3228270

[36] Marantes I, Achenbach SJ, Atkinson EJ, Khosla S, Melton LJ 3rd, Amin S. Is vitamin D a determinant of muscle mass and strength? J Bone Miner Res. 2011;26(12):2860–71. 21915904 10.1002/jbmr.510 PMC3248226

[37] Barker T, Henriksen VT, Martins TB, Hill HR, Kjeldsberg CR, Schneider ED, Dixon BM, Weaver LK. Higher serum 25-hydroxyvitamin D concentrations associate with a faster recovery of skeletal muscle strength after muscular injury. Nutrients. 2013;5(4):1253–75. 23595134 10.3390/nu5041253 PMC3705346

[38] Salacinski AJ, Regueiro MD, Broeder CE, McCrory JL. Decreased neuromuscular function in Crohn’s disease patients is not associated with low serum vitamin D levels. Dig Dis Sci. 2013;58(2):526–33. 22949179 10.1007/s10620-012-2372-4

[39] Barker T, Henriksen VT, Rogers VE, Aguirre D, Trawick RH, Lynn Rasmussen G, Momberger NG. Vitamin D deficiency associates with γ-tocopherol and quadriceps weakness but not inflammatory cytokines in subjects with knee osteoarthritis. Redox Biol. 2014;2:466–74. 24624336 10.1016/j.redox.2014.01.024 PMC3949095

[40] Almurdhi MM, Reeves ND, Bowling FL, Boulton AJ, Jeziorska M, Malik RA. Reduced lower-limb muscle strength and volume in patients with type 2 diabetes in relation to neuropathy, intramuscular fat, and vitamin D levels. Diabetes Care. 2016;39(3):441–7. 26740641 10.2337/dc15-0995 PMC5317239

[41] Jamil NA, Gray SR, Fraser WD, Fielding S, Macdonald HM. The relationship between vitamin D status and muscle strength in young healthy adults from sunny climate countries currently living in the northeast of Scotland. Osteoporos Int. 2017;28(4):1433–43. 28083666 10.1007/s00198-016-3901-3

[42] Wilson-Barnes SL, Hunt JEA, Williams EL, Allison SJ, Wild JJ, Wainwright J, Lanham-New SA, Manders RJF. Seasonal variation in vitamin D status, bone health and athletic performance in competitive university student athletes: a longitudinal study. J Nutr Sci. 2020;9:e8. 32166023 10.1017/jns.2020.1 PMC7054308

[43] Watson EL, Wilkinson TJ, O’Sullivan TF, Baker LA, Gould DW, Xenophontos S, Graham-Brown M, Major R, Jenkinson C, Hewison M, et al. Association between vitamin D deficiency and exercise capacity in patients with CKD, a cross-sectional analysis. J Steroid Biochem Mol Biol. 2021;210:105861. 33675951 10.1016/j.jsbmb.2021.105861

[44] Wilson-Barnes SL, Hunt JEA, Mendis J, Williams EL, King D, Roberts H, Lanham-New SA, Manders RJF. The relationship between vitamin D status, intake and exercise performance in UK University-level athletes and healthy inactive controls. PLoS ONE. 2021;16(4):e0249671. 33798240 10.1371/journal.pone.0249671 PMC8018647

[45] Civelek GM, Pekyavas NO, Cetin N, Cosar SN, Karatas M. Association of vitamin D deficiency with muscle strength and quality of life in postmenopausal women. Climacteric. 2014;17(4):472–7. 24605869 10.3109/13697137.2014.898265

[46] Hamilton B, Whiteley R, Farooq A, Chalabi H. Vitamin D concentration in 342 professional football players and association with lower limb isokinetic function. J Sci Med Sport. 2014;17(1):139–43. 23623203 10.1016/j.jsams.2013.03.006

[47] Yumrutepe T, Aytemur ZA, Baysal O, Taskapan H, Taskapan CM, Hacievliyagil SS. Relationship between vitamin D and lung function, physical performance and balance on patients with stage I-III chronic obstructive pulmonary disease. Rev Assoc Med Bras (1992). 2015;61(2):132–8. 26107362 10.1590/1806-9282.61.02.132

[48] Kara M, Ekiz T, Kara Ö, Tiftik T, Malas F, Özbudak Demir S, Özgirgin N. Does vitamin D affect muscle strength and architecture? An isokinetic and ultrasonographic study. Asia Pac J Clin Nutr. 2017;26(1):85–8. 28049266 10.6133/apjcn.102015.12

[49] Stockton KA, Kandiah DA, Paratz JD, Bennell KL. Fatigue, muscle strength and vitamin D status in women with systemic lupus erythematosus compared with healthy controls. Lupus. 2012;21(3):271–8. 22004972 10.1177/0961203311425530

[50] Balogun S, Aitken D, Winzenberg T, Wills K, Scott D, Callisaya M, Jones G. Longitudinal associations of Serum 25-hydroxyvitamin D, physical activity, and knee pain and dysfunction with muscle loss in community-dwelling older adults. J Gerontol A Biol Sci Med Sci. 2018;73(4):526–31. 28958061 10.1093/gerona/glx157

[51] Brech GC, Ciolac EG, Peterson MD, Greve JM. Serum 25-hydroxyvitamin D levels are associated with functional capacity but not with postural balance in osteoporotic postmenopausal women. Clinics (Sao Paulo). 2017;72(1):11–6. 28226027 10.6061/clinics/2017(01)03 PMC5251195

[52] Kim DK, Park G, Kuo LT, Park WH. Association of vitamin D status with lower limb muscle strength in professional basketball players: a cross-sectional study. Nutrients. 2020;12(9):2715. 32899479 10.3390/nu12092715 PMC7551193

[53] Landis JR, Koch GG. An application of hierarchical kappa-type statistics in the assessment of majority agreement among multiple observers. Biometrics. 1977;33(2):363–74 884196

[54] Holick MF. Vitamin D deficiency. N Engl J Med. 2007;357(3):266–81. 17634462 10.1056/NEJMra070553

[55] Holick MF, Binkley NC, Bischoff-Ferrari HA, Gordon CM, Hanley DA, Heaney RP, Murad MH, Weaver CM. Evaluation, treatment, and prevention of vitamin D deficiency: an Endocrine Society clinical practice guideline. J Clin Endocrinol Metab. 2011;96(7):1911–30. 21646368 10.1210/jc.2011-0385

[56] Society GN. New reference values for vitamin D. Ann Nutr Metab. 2012;60(4):241–6 22677925 10.1159/000337547

[57] Kennel KA, Drake MT, Hurley DL. Vitamin D deficiency in adults: when to test and how to treat. Mayo Clin Proc. 2010;85(8):752–7; quiz 757–8. 20675513 10.4065/mcp.2010.0138 PMC2912737

[58] Rantanen T, Guralnik JM, Ferrucci L, Penninx BW, Leveille S, Sipilä S, Fried LP. Coimpairments as predictors of severe walking disability in older women. J Am Geriatr Soc. 2001;49(1):21–7. 11207838 10.1046/j.1532-5415.2001.49005.x

[59] Balsamo S, da Mota LM, de Carvalho JF, Nascimento Dda C, Tibana RA, de Santana FS, Moreno RL, Gualano B, dos Santos-Neto L. Low dynamic muscle strength and its associations with fatigue, functional performance, and quality of life in premenopausal patients with systemic lupus erythematosus and low disease activity: a case-control study. BMC Musculoskelet Disord. 2013;14:263. 24011222 10.1186/1471-2474-14-263 PMC3847135

[60] Stenholm S, Mehta NK, Elo IT, Heliövaara M, Koskinen S, Aromaa A. Obesity and muscle strength as long-term determinants of all-cause mortality–a 33-year follow-up of the Mini-Finland Health Examination Survey. Int J Obes (Lond). 2014;38(8):1126–32. 24232499 10.1038/ijo.2013.214 PMC4022712

[61] Bischoff-Ferrari HA, Dawson-Hughes B, Willett WC, Staehelin HB, Bazemore MG, Zee RY, Wong JB. Effect of Vitamin D on falls: a meta-analysis. JAMA. 2004;291(16):1999–2006. 15113819 10.1001/jama.291.16.1999

[62] Kalyani RR, Stein B, Valiyil R, Manno R, Maynard JW, Crews DC. Vitamin D treatment for the prevention of falls in older adults: systematic review and meta-analysis. J Am Geriatr Soc. 2010;58(7):1299–310. 20579169 10.1111/j.1532-5415.2010.02949.x PMC3125705

[63] Bischoff-Ferrari HA, Dawson-Hughes B, Staehelin HB, Orav JE, Stuck AE, Theiler R, Wong JB, Egli A, Kiel DP, Henschkowski J. Fall prevention with supplemental and active forms of vitamin D: a meta-analysis of randomised controlled trials. BMJ. 2009;339:b3692. 19797342 10.1136/bmj.b3692 PMC2755728

[64] Moreland JD, Richardson JA, Goldsmith CH, Clase CM. Muscle weakness and falls in older adults: a systematic review and meta-analysis. J Am Geriatr Soc. 2004;52(7):1121–9. 15209650 10.1111/j.1532-5415.2004.52310.x

[65] Scott D, Stuart AL, Kay D, Ebeling PR, Nicholson G, Sanders KM. Investigating the predictive ability of gait speed and quadriceps strength for incident falls in community-dwelling older women at high risk of fracture. Arch Gerontol Geriatr. 2014;58(3):308–13. 24331098 10.1016/j.archger.2013.11.004

[66] Halfon M, Phan O, Teta D. Vitamin D: a review on its effects on muscle strength, the risk of fall, and frailty. Biomed Res Int. 2015;2015:953241. 26000306 10.1155/2015/953241 PMC4427016

[67] Zhang L, Quan M, Cao ZB. Effect of vitamin D supplementation on upper and lower limb muscle strength and muscle power in athletes: A meta-analysis. PLoS ONE. 2019;14(4):e0215826. 31039170 10.1371/journal.pone.0215826 PMC6490896

[68] Beaudart C, Buckinx F, Rabenda V, Gillain S, Cavalier E, Slomian J, Petermans J, Reginster JY, Bruyère O. The effects of vitamin D on skeletal muscle strength, muscle mass, and muscle power: a systematic review and meta-analysis of randomized controlled trials. J Clin Endocrinol Metab. 2014;99(11):4336–45. 25033068 10.1210/jc.2014-1742

[69] Sinha A, Hollingsworth KG, Ball S, Cheetham T. Improving the vitamin D status of vitamin D deficient adults is associated with improved mitochondrial oxidative function in skeletal muscle. J Clin Endocrinol Metab. 2013;98(3):E509–513. 23393184 10.1210/jc.2012-3592

[70] Montenegro KR, Cruzat V, Carlessi R, Newsholme P. Mechanisms of vitamin D action in skeletal muscle. Nutr Res Rev. 2019;32(2):192–204. 31203824 10.1017/S0954422419000064

[71] Srikuea R, Zhang X, Park-Sarge OK, Esser KA. VDR and CYP27B1 are expressed in C2C12 cells and regenerating skeletal muscle: potential role in suppression of myoblast proliferation. Am J Physiol Cell Physiol. 2012;303(4):C396–405. 22648952 10.1152/ajpcell.00014.2012 PMC3422988

[72] Bischoff-Ferrari HA, Borchers M, Gudat F, Dürmüller U, Stähelin HB, Dick W. Vitamin D receptor expression in human muscle tissue decreases with age. J Bone Miner Res. 2004;19(2):265–9. 14969396 10.1359/jbmr.2004.19.2.265

[73] Grundberg E, Brändström H, Ribom EL, Ljunggren O, Mallmin H, Kindmark A. Genetic variation in the human vitamin D receptor is associated with muscle strength, fat mass and body weight in Swedish women. Eur J Endocrinol. 2004;150(3):323–8. 15012617 10.1530/eje.0.1500323

[74] Geusens P, Vandevyver C, Vanhoof J, Cassiman JJ, Boonen S, Raus J. Quadriceps and grip strength are related to vitamin D receptor genotype in elderly nonobese women. J Bone Miner Res. 1997;12(12):2082–8. 9421241 10.1359/jbmr.1997.12.12.2082

[75] Girgis CM, Cha KM, So B, Tsang M, Chen J, Houweling PJ, Schindeler A, Stokes R, Swarbrick MM, Evesson FJ, et al. Mice with myocyte deletion of vitamin D receptor have sarcopenia and impaired muscle function. J Cachexia Sarcopenia Muscle. 2019;10(6):1228–40. 31225722 10.1002/jcsm.12460 PMC6903451

[76] Knutsen KV, Madar AA, Lagerløv P, Brekke M, Raastad T, Stene LC, Meyer HE. Does vitamin D improve muscle strength in adults? A randomized, double-blind, placebo-controlled trial among ethnic minorities in Norway. J Clin Endocrinol Metab. 2014;99(1):194–202. 24248184 10.1210/jc.2013-2647

[77] Kenny AM, Biskup B, Robbins B, Marcella G, Burleson JA. Effects of vitamin D supplementation on strength, physical function, and health perception in older, community-dwelling men. J Am Geriatr Soc. 2003;51(12):1762–7. 14687355 10.1046/j.1532-5415.2003.51561.x

[78] Stockton KA, Mengersen K, Paratz JD, Kandiah D, Bennell KL. Effect of vitamin D supplementation on muscle strength: a systematic review and meta-analysis. Osteoporos Int. 2011;22(3):859–71. 20924748 10.1007/s00198-010-1407-y

[79] Rosendahl-Riise H, Spielau U, Ranhoff AH, Gudbrandsen OA, Dierkes J. Vitamin D supplementation and its influence on muscle strength and mobility in community-dwelling older persons: a systematic review and meta-analysis. J Hum Nutr Diet. 2017;30(1):3–15. 27460044 10.1111/jhn.12394 PMC5248635

[80] Eyles DW, Smith S, Kinobe R, Hewison M, McGrath JJ. Distribution of the vitamin D receptor and 1 alpha-hydroxylase in human brain. J Chem Neuroanat. 2005;29(1):21–30. 15589699 10.1016/j.jchemneu.2004.08.006

[81] Tomlinson PB, Joseph C, Angioi M. Effects of vitamin D supplementation on upper and lower body muscle strength levels in healthy individuals. A systematic review with meta-analysis. J Sci Med Sport. 2015;18(5):575–80. 25156880 10.1016/j.jsams.2014.07.022

[82] Delp MD, Duan C. Composition and size of type I, IIA, IID/X, and IIB fibers and citrate synthase activity of rat muscle. J Appl Physiol (1985). 1996;80(1):261–70. 8847313 10.1152/jappl.1996.80.1.261

[83] Barker T, Martins TB, Kjeldsberg CR, Trawick RH, Hill HR. Circulating interferon-γ correlates with 1,25(OH)D and the 1,25(OH)D-to-25(OH)D ratio. Cytokine. 2012;60(1):23–6. 22704696 10.1016/j.cyto.2012.05.015

[84] Sanders KM, Stuart AL, Williamson EJ, Simpson JA, Kotowicz MA, Young D, Nicholson GC. Annual high-dose oral vitamin D and falls and fractures in older women: a randomized controlled trial. JAMA. 2010;303(18):1815–22. 20460620 10.1001/jama.2010.594

[85] Smith H, Anderson F, Raphael H, Maslin P, Crozier S, Cooper C. Effect of annual intramuscular vitamin D on fracture risk in elderly men and women–a population-based, randomized, double-blind, placebo-controlled trial. Rheumatology (Oxford). 2007;46(12):1852–7. 17998225 10.1093/rheumatology/kem240

[86] Trivedi DP, Doll R, Khaw KT. Effect of four monthly oral vitamin D3 (cholecalciferol) supplementation on fractures and mortality in men and women living in the community: randomised double blind controlled trial. BMJ. 2003;326(7387):469. 12609940 10.1136/bmj.326.7387.469 PMC150177

[87] Ozturk GTUM, Ozturk Y, Inanir A. Evaluation of muscle performance in patients with vitamin d deficiency: Preliminary study. Turk Osteoporoz Dergisi. 2013;19(1):17–9.

