# Supplementary material for: Effect of serum 25-hydroxyvitamin D level on quadriceps strength: a systematic review and meta-analysis
Source: BMC Sports Sci Med Rehabil. 2024 Oct 14;16:215. doi: 10.1186/s13102-024-01007-z (PMC11476103; doi:10.1186/s13102-024-01007-z)
Supplement: Supplementary file 1 — Supplementary Material 1. [file 13102_2024_1007_MOESM1_ESM.docx]

| **Supplementary Table 1**: Search Strategy | | | | | |
| --- | --- | --- | --- | --- | --- |
|  | Searches | *PubMed* | *Ovid* | *CINAHL*  (EBSCOhost) | *SPORTDiscus*  (EBSCOhost) |
| S1 | vitamin D or D vitamin or ergocalciferol* or cholecalciferol* or 25OHD* or calcifediol* or calcidiol* or 25-hydroxycholecalciferol* or 25-hydroxyvitamin D* | 96,141 | 152846 | 29,575 | 4,097 |
| S2 | isokinetic or isometric or CSA or muscle cross section or physiological cross section or quadricep cross section or muscle size or muscle volume | 637,738 | 93,651 | 21,538 | 23,652 |
| S3 | lower limb or lower body or leg or quadricep* or quad* muscle or rectus femoris or vastus  lateral* or vastus externus or VL or vastus medial* or vastus internus or teardrop muscle or VMO or vastus intermedius or Cruraeus or knee or knee extensor or proximal | 940,419 | 860,518 | 185,848 | 108,073 |
| S4 | dynamometer* or torque or strength or voluntary contraction or MVC or knee extension or quadricep* concentric or angular velocit* or angular speed or CSA or muscle cross section  or physiological cross section or quadricep cross section or muscle size or muscle volume | 590,648 | 532,004 | 118,421 | 104,608 |
| S5 | S1 and S2 and S3 and S4 | 397 | 168 | 52 | 22 |
