# Supplementary material for: Effect of serum 25-hydroxyvitamin D level on quadriceps strength: a systematic review and meta-analysis
Source: BMC Sports Sci Med Rehabil. 2024 Oct 14;16:215. doi: 10.1186/s13102-024-01007-z (PMC11476103; doi:10.1186/s13102-024-01007-z)
Supplement: Supplementary file 2 — Supplementary Material 2. [file 13102_2024_1007_MOESM2_ESM.docx]

| **Supplementary table 2**: PICOS inclusion and exclusion criteria for study selection. | | |
| --- | --- | --- |
| **Domain** | **Inclusion Criteria** | **Exclusion Criteria** |
| **Population** | Clinical study conducted on human populations regardless of age, gender, ethnicity, presenting complaint. | *In vivo* studies  *Ex vivo* studies  Animal studies |
| **Intervention** | Studies that measure serum concentration of 25OHD as an indicator of vitamin D status. | Studies using other types of indicators for vitamin D status such as 1,25- dihydroxyvitamin D, vitamin D receptor density, or dietary intake of vitamin D. |
| **Comparison** | N/A | N/A |
| **Outcome** | Studies reporting either isometric measurements at any knee flexion angle, isokinetic measurements at any angular velocity, quadriceps maximal voluntary contraction, or quadriceps muscle size. | Studies where no outcome measures are directly related to quadriceps function |
| **Study Type** | Primary studies written in English with full-text available | Case reports, abstracts, reviews.  Studies not written in English. |
