# Supplementary material for: Effect of serum 25-hydroxyvitamin D level on quadriceps strength: a systematic review and meta-analysis
Source: BMC Sports Sci Med Rehabil. 2024 Oct 14;16:215. doi: 10.1186/s13102-024-01007-z (PMC11476103; doi:10.1186/s13102-024-01007-z)
Supplement: Supplementary file 3 — Supplementary Material 3. [file 13102_2024_1007_MOESM3_ESM.docx]

**Supplementary Table 3:** Outcome Measurements

| **Study** | **Measurement Protocol** | **Model** | **Outcome** | **Association with 25OHD** |
| --- | --- | --- | --- | --- |
| **ISOKINETIC** | | | | |
| **Isokinetic at 60˚/s** | | | | |
| Grimaldi et al., 2013 | Dominant knee average peak torque, 4 maximal continuous contractions | Biodex System 3 | Total: 137 Nm  Men: 177 Nm  Women: 107 Nm | between-subject effects (Age and Gender were controlled):  F = 2.321 (p = 0.13) |
| Barker et al., 2014 | Quadriceps peak torque, 6 contractions,  healthy knee prior to symptomatic knee | Biodex S4 | Symptomatic acceleration (m/s), Deficient/ Insufficient/ Sufficient:  62.4 ± 7.0/ 56.3 ± 5.7/ 52.8 ± 4.4  Non-symptomatic acceleration (m/s), Deficient/ Insufficient/ Sufficient:  70.6 ± 9.7/ 54.3 ± 5.2/ 43.3 ± 4.6 | Knee extension peak torque (Nm) was significantly decreased in the vitamin D ‘Deficient’ group: (p < 0.05) |
| Civelek et al., 2014 | Knee extensor muscle strength L, 5 consecutive concentric motions | Cybex 770 Norm | Appendix F1 | Between vitamin D  deficient and normal groups: (p = 0.561) |

| Outcome Measurements (Cont.) | | | | |
| --- | --- | --- | --- | --- |
| **Isokinetic at 60˚/s** | | | | |
| Civelek et al., 2014 | Knee extensor muscle strength R, 5 consecutive concentric motions | Cybex 770 Norm | Appendix F1 | Between vitamin D  deficient and normal groups: (p = 0.139) |
| Hamilton et al., 2014 | Knee isokinetic extension peak torque L, 5 repetitions,  Left then right leg | Biodex 3.0 system | <10/ 10-20/ 20-30/ >30 ng/mL (Nm):  224.8 ± 5.9/ 235.4 ± 4.0/  238.1 ± 4.7/ 235.4 ± 5.2 | Between-group: (p = 0.465) |
|  | Knee isokinetic extension peak torque R, 5 repetitions,  Left then right leg |  | <10/ 10-20/ 20-30/ >30 ng/mL (Nm):  223.7 ± 6.0/ 228.3 ± 3.8/  227.9 ± 4.4/ 224.8 ± 4.1 | Between-group: (p = 0.906) |
| Kara et al., 2017 | Peak torque/body weight of the knee muscles,  3 maximal reciprocal contractions | Biodex System 3 (Pro Multi-  joint) | Appendix F1 | Between-group: (p = 0.038) |
| Książek et  al., 2018 | Knee extension concentric R,  5 repetitions | Biodex  Multi-Joint 4 | 212.7 ± 28.5 Nm | r = 0.39 |
|  | Knee extension concentric L,  5 repetitions |  | 211.3 ± 24.3 Nm | r = 0.37 |

| Outcome Measurements (Cont.) | | | | |
| --- | --- | --- | --- | --- |
| **Isokinetic at 60˚/s** |  |  |  |  |
| Kim et al., 2020 | Dominant knee extension strength, Maximum peak torque recorded | CSMI  Medical Solutions | Deficiency: 165.3 ± 33.0 Nm  Insufficiency: 172.3 ± 22.7 Nm  Sufficiency: 166.2 ± 20.5 Nm | r = 0.134 (p = 0.436)  Between-group: (p = 0.753) |
|  | Non-dominant knee extension strength, Maximum peak torque recorded |  | Deficiency: 160.1 ± 30.3 Nm  Insufficiency: 173.3 ± 25.2 Nm  Sufficiency: 154.2 ± 21.5 Nm | r = -0.058 (p = 0.737)  Between-group: (p = 0.182) |
| **Isokinetic at 90˚/s** |  |  |  |  |
| Yumrutepe et al., 2015 | Dominant extensor muscles, 10 repetitions per minute | Biodex System 3 Pro | COPD: 92.1 ± 32.7 Nm  <15 ng/mL: 85.0 ± 29.7 Nm  >15 ng/mL: 102.9 ± 34.5 Nm)  Control: 106.1 ± 33.0 Nm | In COPD patients, r = 0.254 (p = 0.016)  Between 25(OH)D <15 ng/mL and >15 ng/mL:  (p = 0.01) |
| Brannstrom et al., 2017 | Bilateral knee extension, 5 consecutive repetitions | Biodex 3 system | 139.0 ± 15.5 Nm  Extension/body weight:  244.4 ± 23.7 % | Knee extension peak  torque/body weight: (r = 0.181) |

| Outcome Measurements (Cont.) | | | | |
| --- | --- | --- | --- | --- |
| **Isokinetic at 120˚/s** | | | | |
| Civelek et al., 2014 | Knee extensor muscle strength L, 5 consecutive concentric motions | Cybex 770 Norm | Appendix F1 | Between vitamin D  deficient and normal groups: (p = 0.406) |
|  | Knee extensor muscle strength R, 5 consecutive concentric motions |  |  | Between vitamin D deficient and normal  groups: (p = 0.465) |
| **Isokinetic at 180˚/s** | | | | |
| Grimaldi et al., 2013 | Dominant knee average peak torque, 4 maximal continuous contractions | Biodex System 3 | Total: 92 Nm  Men: 121 Nm  Women: 71 Nm | Between-subject effects (Age and Gender were controlled):  F = 2.468 (p = 0.12) |
| Civelek et al., 2014 | Knee extensor muscle strength L, 5 consecutive concentric motions | Cybex 770 Norm | Appendix F1 | Between vitamin D deficient and normal  groups: (p = 0.741) |
|  | Knee extensor muscle strength R, 5 consecutive concentric motions |  |  | Between vitamin D  deficient and normal groups: (p = 0.452) |

| Outcome Measurements (Cont.) | | | | |
| --- | --- | --- | --- | --- |
| **Isokinetic at 180˚/s** | | | | |
| Yumrutepe et al., 2015 | Dominant extensor muscles, 10 repetitions per minute | Biodex System 3 Pro | COPD: 58.0 ± 17.8 Nm  <15 ng/mL: 55 ± 16.9 Nm  >15 ng/mL: 62.5 ± 18.5 Nm  Control: 68.6 ± 19.6 Nm | In COPD patients,  r = 0.183 (p = 0.084),  Between 25(OH)D <15  ng/mL and >15 ng/mL: (p = 0.04) |
| Kara et al., 2017 | Peak torque/body weight of the knee muscles,  10 maximal reciprocal contractions | Biodex System 3 (Pro Multi- joint) | Group 1: 81.9 ± 13.1 Nm/kg  Group 2: 99.6 ± 13.5 Nm/kg | Between-group: (p = 0.001) |
| Książek et al., 2018 | Knee extension concentric R, 5 repetitions | Biodex Multi-Joint 4 | 96.6 ± 14.3 Nm | r = 0.39 |
|  | Knee extension concentric L,  5 repetitions |  | 93.5 ± 12.4 Nm | r = 0.33 |

| Outcome Measurements (Cont.) | | | | |
| --- | --- | --- | --- | --- |
| **Isokinetic at 300˚/s** | | | | |
| Hamilton et al., 2014 | Knee isokinetic extension peak torque L, 5 repetitions,  Left then right leg | Biodex 3.0 system | <10/ 10-20/ 20-30/ >30 ng/mL (Nm):  123.6 ± 3.9/ 132.2 ± 2.3/  138.0 ± 2.9/ 139.6 ± 3.7 | Between-group: (p = 0.021) |
|  | Knee isokinetic extension peak torque R, 5 repetitions,  Left then right leg |  | <10/ 10-20/ 20-30/ >30 ng/mL (Nm):  122.9 ± 3.7/ 127.7 ± 2.1 /  131.2 ± 2.7/ 128.5 ± 2.9 | Between-group: (p = 0.358) |
| **Isokinetic (angular velocity not reported)** | | | | |
| Brech et al., 2017 | Concentric extension of dominant and non-dominant leg knee joint,  5 maximal repetitions, starting with the dominant limb | Biodex® Multi-joint System 3 | G1/ G2/ G3/ G4 (%): Dominant, 150.3 ± 27.8/ 152.8 ± 27.9/  148.2 ± 34.4/ 158.8 ± 29.5  Non-dom., 146.5 ± 30.0/ 152.9 ± 20.2/  149.4 ± 24.5/ 152.4 ± 29.8 | Normalized peak torque of the knee extensors:  β = 0.59 (p = 0.04) |

| Outcome Measurements (Cont.) | | | | |
| --- | --- | --- | --- | --- |
| **ISOMETRIC** | | | | |
| **Isometric at 45˚ knee flexion** | | | | |
| Salacinski et al., 2013 | Maximal isometric strength of the knee extensors on the subject’s left side,  3 repetitions/ 5-s each,  30-s rest interval between contractions | Customized strength- testing device | Extension peak torque normalized to bodyweight,  ≥40 ng/dL: 0.088 ± 0.007 Nm/kg  <32 ng/dL: 0.057 ± 0.006 Nm/kg  Extension average torque normalized to bodyweight,  ≥40 ng/dL: 0.064 ± 0.006 Nm/kg  <32 ng/dL: 0.043 ± 0.004 Nm/kg | High serum vitamin D levels (>=40 ng/dL) compared with low levels (<32 ng/dL):  Extension peak torque normalized to body weight (p = 0.045); Extension average torque normalized to body  weight (p = 0.014) |

| Outcome Measurements (Cont.) | | | | |
| --- | --- | --- | --- | --- |
| **Isometric at 60˚ knee flexion** | | | | |
| Dretakis et ak., 2010 | Quadriceps muscle strength R,  3 maximal contraction, 15-s each, with 15-s intermediate relaxation | Apparatus’ chair (Cybex) | Female: 115.23 ± 24.74 Nm  Male: 156.38 ± 31.23 Nm | r = 0.491 (p < 0.0005) |
|  | Quadriceps muscle strength L, 3 maximal contraction, 15-s each,  with 15-s intermediate relaxation |  | Female: 112.03 ± 22.86 Nm  Male: 154.60 ± 30.48 Nm | r = 0.496 (p < 0.0005) |
| Rolighed et al., 2014 | Maximum isometric muscle strength, Best from 3 repetitions,  with 30-s brakes | Adjustable dynamometer chair connected to  a computer | Median and 25–75 percentiles, All PHPT: 319 (229 – 373)  Matched controls: 410 (332 - 503)  Asymptomatic PHPT: 370 (278 – 412)  Matched controls: 515 (377 – 578) | Correlation coefficients:  -1.1 (p > 0.05) |
| Salminen et al., 2015 | Maximal isometric muscle strength R, 3-4 maximal efforts,  3-s each, Separated by 30-s of rest | Good Strength | Right knee, 317.3 ± 108.4 N  <50 nmol/L, 297.5 ± 98.6 N  50 - 74.9 nmol/, 318.8 ± 112.2 N  >=75 nmol/L, 329.1 ± 107.1 N | Between-group, Baseline (p = 0.072); Adjusted changes in physical performance  during a one-year follow-  up (p = 0.019) |

| Outcome Measurements (Cont.) | | | | |
| --- | --- | --- | --- | --- |
| **Isometric at 60˚ knee flexion** | | | | |
| Salminen et al., 2015 | Maximal isometric muscle strength L, 3-4 maximal efforts,  3-s each, Separated by 30-s of rest | Good Strength | Left knee, 311.8 ± 105.3 N  <50 nmol/L, 293.7 ± 101.0 N  50 - 74.9 nmol/L, 313.1 ± 108.3 N  >=75 nmol/L, 322.5 ± 101.9 N | Between-group, Baseline (p = 0.096); Adjusted changes in physical performance  during a one-year follow-  up (p = 0.022) |
| **Isometric at 90˚ knee flexion** | | | | |
| Dretakis et al., 2010 | Quadriceps muscle strength R,  3 maximal contraction, 15-s each, with 15-s intermediate relaxation | Apparatus’ chair (Cybex) | Female: 113.00 ± 23.73 Nm  Male: 153.00 ± 34.77 Nm | r = 0.517 (p < 0.0005) |
|  | Quadriceps muscle strength L, 3 maximal contraction, 15-s each,  with 15-s intermediate relaxation |  | Female: 116.14 ± 26.25 Nm  Male: 167.46 ± 43.45 Nm | r = 0.438 (p = 0.002) |

| Outcome Measurements (Cont.) | | | | |
| --- | --- | --- | --- | --- |
| **Isometric at 90˚ knee flexion** | | | | |
| Marantes et | Isometric knee extension force, | Custom-built | Men/ Women (Nm), | Between-group, |
| al., 2011 | 3 trials, 3-5-s each, | dynamometer | Total: 172.2 ± 58.7/ 93.7 ± 28.8 | Men (p = 0.295) |
|  | 1-2 minutes rest in-between | chair | 5-16 ng/mL: 161.44/ 89.97 | Women (p = 0.301) |
|  |  |  | 17-21 ng/mL: 175.58/ 94.48 |  |
|  |  |  | 22-27 ng/mL: 178.75/ 98.63 |  |
|  |  |  | 28-68 ng/mL: 170.79/ 91.93 |  |
| Rolighed et | Maximum isometric muscle strength, | Adjustable | Median and 25–75 percentiles: | Correlation coefficients: |
| al., 2014 | Best from 3 repetitions, | dynamometer | All PHPT: 320 (249 – 392) | -1.1 (p > 0.05) |
|  | with 30-s brakes | chair | Matched controls: 358 (289 - 466) |  |
|  |  | connected to | Asymptomatic PHPT: 381 (279 – 459) |  |
|  |  | a computer | Matched controls: 504 (338 – 591) |  |
| Wilson-Barnes | Peak isometric knee extensor torque, | CSMI Humac | Autumn (Nm), | Autumn, |
| et al., 2020 | 3 repetitions, | Norm | <25 nmol/L: 236.9 ± 32.0 | >75 nmol/L vitamin D |
|  | 5-s each, |  | 25 – 50 nmol/L: 227.8 ± 61.4 | status superior to vitamin |
|  | separated by a 1-min rest |  | 50 -75 nmol/L: 243.6 ± 73.2 | D-insufficient (25–50 |
|  |  |  | >75 nmol/L: 317.3 ± 114. | nmol/l) outcomes |
|  |  |  |  | (p = 0·019) |

| Outcome Measurements (Cont.) | | | | |
| --- | --- | --- | --- | --- |
| **Isometric at 90˚ knee flexion** | | | | |
| Wilson-Barnes et al., 2021 | Peak isometric knee extensor torque with their non-dominant leg, highest of 3 repetitions, 5-s each,  separated by a 1-min rest | CSMI Humac Norm | <50/ >50 nmol/L (Nm): Spring, 213.6 ± 71.5/ 215.1 ± 52.5  Summer: 202.0 ± 72.1/ 240.7 ± 80.6 | Between vitamin D status and sport participation, Summer: (p = 0.3) |
| **Isometric at 110˚ knee flexion** | | | | |
| Grimaldi et  al., 2013 | Knee extension Average Peak Torque,  3 maximal contractions | Biodex  System 3 | Total/ Men/ Women (Nm):  178/ 227/ 141 | Age-/ Gender-controlled:  F = 4.62 (p = 0.03) |
| **Isometric at 115˚ knee flexion** | | | | |
| Balogun et al., 2018 | Lower limb muscle strength, Mean score of 2 trails | TTM  Muscular Meter | Lost to follow up compared with completed the 10-year follow-up assessment at baseline,  87.7 ± 47.0 vs 96.9 ± 50.5 kg | Between-person effect: β = 0.16 (0.05, 0.27);  Within-person effect:  β = 0.10 (0.03, 0.17) |
| **Isometric (degree of knee flexion not reported)** | | | | |
| Zamboni et  al., 2002 | Isometric strength of the dominant knee,  MEN, highest peak torque in 3 trials | Model 160 | 18.0 ± 5.8 kg | r = -0.01 (p > 0.05) |
|  | Isometric strength of the dominant knee,  WOMEN, highest peak torque in 3 trials |  | 10.4 ± 4.5 kg | r = 0.17 (p < 0.05) |

| Outcome Measurements (Cont.) | | | | |
| --- | --- | --- | --- | --- |
| **Isometric (degree of knee flexion not reported)** | | | | |
| Houston et al., 2011 | Maximum strength of the knee extensor muscles,  recorded during a 4-s effort | Litek Isometric Chair | Knee extensor strength adjusted for body size (Nm),  <20 ng/mL: 70.2 ± 2.6  20 - <30 ng/mL: 72.9 ± 2.5  >= 30 ng/mL: 73.9 ± 2.6 | Between-group (adjusted for body size): (p = 0.12); Significantly lower (compared deficient and sufficient 25OHD levels), adjusted season and sociodemographic  characteristics: (p <0.05) |
| Stockton et al., 2012 | Isometric muscle strength of knee extensors of dominant limbs, best of 3 attempts was recorded | Lafayette Nicholas Manual Muscle Tester model  01163 | SLE, 51.9 ± 12.5 Nm  Control, 63.0 ± 15.6 Nm | No significant correlation |

| Outcome Measurements (Cont.) | | | | |
| --- | --- | --- | --- | --- |
| **Isometric (degree of knee flexion not reported)** | | | | |
| Barker et al., 2013 | Peak knee isometric contraction on each leg IMMIDIATE post- exercise,  3 repetitions, 3-s each,  separated by 1 min of rest | Horizontal Plyo-Press | Appendix F2 | Coefficient, standardized:  -0.68 (p = 0.03) |
|  | Peak knee isometric contraction on each leg 24-H after exercise,  3 repetitions, 3-s each,  separated by 1 min of rest |  |  | Coefficient, standardized:  -0.52 (p = 0.14) |
|  | Peak knee isometric contraction on each leg 48-H after exercise,  3 repetitions, 3-s each,  separated by 1 min of rest |  |  | Coefficient, standardized:  -0.68 (p = 0.04) |
|  | Peak knee isometric contraction on each leg 72-H after exercise,  3 repetitions, 3-s each,  separated by 1 min of rest |  |  | Coefficient, standardized:  -0.63 (p = 0.04) |

| Outcome Measurements (Cont.) | | | | | |
| --- | --- | --- | --- | --- | --- |
| **Maximal Voluntary Contraction** | | | | |  |
| Dhesi et al., 2002 | Isometric quadriceps strength and activation of dominant R,  assessed using a strain gauge system, strongest among 3 MVC | A specially constructed chair | Appendix F3 | Group 1 tended to be weaker than patients with 25OHD >12 μg/L  (Groups 2 and 3) (196N vs. 236N,  t = 1.99, p = 0.051) | |
| Annweiler et al., 2009 | Maximal isometric voluntary contraction strength of dominant and non-dominant, assessed using a strain gauge system, highest value of 3 MVC strength for  dominant and non-dominant leg | Computerized dynamometers | 175.1 ± 52.9 N/m^2^  <15 ng/mL: 174.9 ± 53.2 N/m^2^  15-30 ng/mL: 175.9 ± 52.6 N/m^2^  >30 ng/mL: 173.4 ± 53.1 N/m^2^ | Coefficient of regression beta (95% CI): crude β =  0.03 (-0.34;0.39, p =  0.891); adjusted β = -0.04  (-0.38;0.31, p = 0.837) | |
| Almurdhi et al., 2016 | Maximal voluntary isometric contractions of the knee extensors,  at 85˚,70˚, 55˚ of knee flexion,  2-min rest interval | Cybex NORM | <25 nmol/L, 1.2 ± 0.1 Nm/kg  >25 nmol/L, 1.3 ± 0.5 Nm/kg | Between-group: (p = 0.32) | |

| Outcome Measurements (Cont.) | | | | |
| --- | --- | --- | --- | --- |
| **Maximal Voluntary Contraction** | | | | |
| Jamil et al., 2017 | Maximal isometric strength of the knee extensor muscles R during an MVC, repeated a minimum of 3 times,  over 10-s each,  120-s rest in-between | Biodex dynamometer System 2 | 130.9 ± 39.7 Nm | Adjusted of VDBP: (Estimate = 0.01;  SEM = 0.01; p = 0.049;  95% CI: 0.0001, 0.06) |
| **Muscle Size** | | | | |
| Bredella et al., 2011 | Cross-sectional CT of the left mid-thigh, single 1 cm axial image of the mid left thigh was obtained, | Quantitative CT | Thigh muscle CSA: 147.2 ± 19.0 cm^2^ Thigh muscle density: 46.8 ± 5.3 HU | No significant association between 25(OH)D and thigh muscle CSA (p = 0.8) or density (p = 0.1) |
| Almurdhi et al., 2016 | Serial CSAs of the knee extensors (vastus medialis, vastus intermedius, vastus lateralis, rectus femoris) | Heidelberg Retina Tomograph III Rostock  Cornea Mod. | Muscle volume:  <25 nmol/L, 932.1 ± 427.3 cm^3^  >25 nmol/L, 1,122.7 ± 175.1 cm^3^ | Between-group: (p = 0.18) |

| Outcome Measurements (Cont.) | | | | |
| --- | --- | --- | --- | --- |
|  |  | **Muscle Size** |  |  |
| Kara et al., 2017 | Ultrasonographic measurement on Vastus lateralis from the mid-thigh level | 7-12 MHz  linear probe | Muscle thickness, Group 1, 8.07 ± 1.58 mm  Group II, 7.60 ± 1.68 mm Fascicle length,  Group 1, 55.6 ± 13.0 mm  Group II, 48.0 ± 13.7mm | Between-groups: Muscle thickness (p = 0.450)  Fascicle length (p = 0.144) |
| Watson et al., 2021 | (I) Quadriceps volume R,  3 T Siemens Skyra HD MRI scanner, axial plane, T1 turbo spin-echo sequence  (II) Rectus femoris R cross-sectional area | (I) MRI  (II) 2-D  B-mode ultrasound | Muscle volume, Full Cohort (n = 33):  931.7 cm^3^ (752.5 – 1091.9);  Rectus femoris CSA, Full Cohort (n = 36):  8.2 cm^2^ (5.7 – 10.2) | Bivariate correlations between 25(OH)D and, RF-CSA  (p = 0.16)  Quadriceps volume (p = 0.89) |
