# Supplementary material for: Effect of serum 25-hydroxyvitamin D level on quadriceps strength: a systematic review and meta-analysis
Source: BMC Sports Sci Med Rehabil. 2024 Oct 14;16:215. doi: 10.1186/s13102-024-01007-z (PMC11476103; doi:10.1186/s13102-024-01007-z)
Supplement: Supplementary file 4 — Supplementary Material 4. [file 13102_2024_1007_MOESM4_ESM.docx]

| **Supplementary Table 4**: GRADE Quality of Evidence | | | | | | | |
| --- | --- | --- | --- | --- | --- | --- | --- |
| *Correlation between serum 25OHD levels and:* | Study Limitations | Consistency | Directness | Precision | Publication Bias | Quality* | |
| IK measurement in any angular velocities | No limitations | No important inconsistency | Direct | No important imprecision | Unlikely | Low  ⊕⊕ΟΟ | |
| IM measurement in any angle of knee flexion | No limitations | Inconsistent (-1) | Direct | No important imprecision | Unlikely | Very Low  ⊕ΟΟΟ | |
| MVC measurement | Serious limitations (−1) | No important inconsistency | Direct | Imprecise (-1) | Likely (-1) | Very Low  ⊕ΟΟΟ | |
| IK measurement in athletic populations | No limitations | No important inconsistency | Direct | No important imprecision | Unlikely | Low  ⊕⊕ΟΟ | |
| IM measurement in elderly populations | No limitations | Inconsistent (-1) | Direct | No important imprecision | Likely (-1) | Very Low  ⊕ΟΟΟ | |
| IK measurement at 60^o^/s | No limitations | No important inconsistency | Direct | Imprecise (-1) | Likely (-1) | Very Low  ⊕ΟΟΟ | |
| IK measurement at 180^o^/s | No limitations | No important inconsistency | Direct | No important imprecision | Unlikely | Low  ⊕⊕ΟΟ | |
| *Baseline quality of evidence for non-RCT studies is ‘low’.  IK: isokinetic; IM: isometric; MVC: maximal voluntary contraction; CI: confidence interval | | | | | | |  |
