# Supplementary material for: Effect of serum 25-hydroxyvitamin D level on quadriceps strength: a systematic review and meta-analysis
Source: BMC Sports Sci Med Rehabil. 2024 Oct 14;16:215. doi: 10.1186/s13102-024-01007-z (PMC11476103; doi:10.1186/s13102-024-01007-z)
Supplement: Supplementary file 5 — Supplementary Material 5. [file 13102_2024_1007_MOESM5_ESM.docx]

**Supplementary Table 6:** Quality in Prognosis Studies (QUIPs)^46^

| Author and Year | Assessment | Study Participation | Study Attrition | Prognostic  Factor Measurement | Outcome Measurement | Study Cofounding | Statistical  Analysis and Reporting | Overall Rating |
| --- | --- | --- | --- | --- | --- | --- | --- | --- |
| Jamil et al.,  2017 | MVC | Low | Low | Low | Low | Low | Low | LOW |
| Balogun et al.,  2018 | IM | Low | High | Moderate | Low | Low | Moderate | MODERATE |
| Wilson-Barnes et  al., 2020 | IM | Low | Low | Low | Low | Low | Low | LOW |
| Wilson-Barnes et  al., 2021 | IM | Low | Low | Low | Low | Low | Low | LOW |
| Overall rating  per domain |  | LOW | MODERATE | LOW | LOW | LOW | LOW |  |
